# Effect of Long-Range Transported Aerosol on Urban Air Quality in Eastern Germany

**DOI:** 10.1021/acsestair.5c00126

**Published:** 2025-07-30

**Authors:** Samira Atabakhsh, Laurent Poulain, Mira Pöhlker, Khanneh Wadinga Fomba, Hartmut Herrmann

**Affiliations:** † Atmospheric Chemistry Department (ACD), 28397 Leibniz Institute for Tropospheric Research (TROPOS), Leipzig 04318, Germany; ‡ Atmospheric Microphysics Department (AMP), Leibniz Institute for Tropospheric Research (TROPOS), Leipzig 04318, Germany

**Keywords:** submicron particle (PM_1_), source apportionment, air quality, AMS, ACSM, urban increment, long-range transport (LRT)

## Abstract

Submicron particles (PM_1_) play a crucial role in air quality and human health. This study investigates the influence of long-range transport (LRT) on urban aerosol levels in Leipzig, Germany, using high-resolution aerosol mass spectrometry measurements at two sites: an urban traffic site (Eisenbahnstrasse, Eiba) and a rural background site (Melpitz), located ∼50 km apart. The sites were analyzed during winter 2017 under two dominant wind regimes: East and West. These sites were directly linked to each other, which was supported by cross-correlation analysis, with a typical time lag of −2 h in East and +4 h in West. Eastern winds brought higher concentrations (Melpitz: 35.50 μg m^–3^, Eiba: 37.47 μg m^–3^), while Western winds led to cleaner conditions. After being corrected for time lag, the Urban Increment (UI) was estimated, showing that during Eastern wind, only ∼9% of PM_1_ mass measured at Eiba was attributed to urban sources, highlighting the dominant contribution of regionally transported aerosol. Furthermore, source apportionment of organic aerosol (OA) identified five major factors—three primary OA and two oxygenated OA—at both sites. The findings underscore the significant role of regional pollution in shaping urban air quality and the need for cross-border emission reduction strategies.

## Graphical abstract

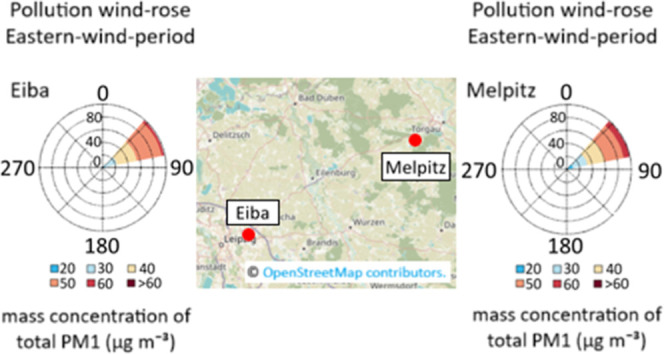

## 1 Introduction

Atmospheric particulate matter (PM) exhibits diverse physical and chemical properties due to its complex and multisource origins. These particles contribute to major environmental and public health challenges, including climate forcing, air quality degradation, and adverse health outcomes.^1^ Globally, air pollution remains a leading cause of premature mortality, with exposure to fine particulate matter (PM_2.5_) responsible for over 400,000 premature deaths annually across Europe.^2^ Despite regulatory efforts by the European Union aimed at reducing PM levels and improving air quality,^3^ substantial uncertainties persist regarding the quantitative links between PM exposure, source identification, and health impacts, which complicate the development of targeted and effective mitigation strategies.^4,5^

In urban areas, PM exposure results from a combination of local emissions, secondary aerosol formation, regional background pollution, and long-range transport (LRT). While extensive research has explored PM chemical composition and source contributions (e.g.,^6,7^ ), quantitative, time-resolved assessments of LRT and background aerosol impacts, particularly based on synchronized multisite measurements, remain limited.^8^ Existing studies highlight the significant influence of LRT on urban PM levels across South America, Asia, and Europe, especially during pollution episodes driven by specific meteorological conditions.^9–11^

Although many of these studies have focused on PM_2.5_, submicron particles (PM_1_) have received growing attention due to their critical role in human health, visibility reduction, and climate impacts.^12–14^ Organic aerosol (OA), a major PM_1_ component, remains particularly challenging to characterize due to its complex seasonal variability and dependence on both primary emissions and atmospheric chemical processes influenced by meteorological conditions and geographical locations.

Advanced online techniques, such as Aerosol Mass Spectrometers (AMS^15^) and Aerosol Chemical Speciation Monitors (ACSM^16^), provide the high-time-resolution necessary to track OA dynamics and PM_1_ composition. Numerous studies have applied these instruments to urban environments, focusing largely on site-specific emission sources and chemical characteristics. Notable examples include the 22 year AMS and ACSM data set from Europe,^7^ the MILAGRO campaign in Mexico City,^17^ and long-term ACSM studies in Helsinki.^18^ However, few studies have leveraged synchronized, multisite online data sets to investigate how transported PM_1_ and OA influence urban air quality, despite their importance for understanding regional source dynamics and improving air quality management.

This study addresses this gap by utilizing a unique, high-time-resolution data set comprising simultaneous AMS and ACSM measurements at two nearby locations: a street canyon station in Leipzig, and the rural background site Melpitz. Building upon the classical urban increment (UI) approach, also known as the Lenschow method,^19^ the method was refined by applying time-lag correction based on cross-correlation analysis, accounting for transport delays between the sites. This enhancement enables not only accurate quantification of the UI but also assessment of its diurnal variability, providing new insights into the temporal dynamics of urban emissions and regional background influences. The data set reveals distinct compositional contrasts between the sites and highlights the critical role of air mass transport in shaping PM_1_ levels in Leipzig. The present work demonstrates the analytical potential of high-time-resolution, multisite online measurements and proposes a scalable framework for future assessments of LRT impacts on urban air quality.

## 2 Materials and Methods

### 2.1 Measuring Sites

Particle sampling was performed at two parallel sites in Eastern Germany: Leipzig Eisenbahnstrasse (Eiba) and the research station of Leibniz Institute for Tropospheric Research (TROPOS) in Melpitz (Mel), approximately 50 km apart, as shown in Figure 1. The site Eiba (51°20′45″ N, 12°24′23″ E, 120 m a.s.l.) is the TROPOS street canyon site that has been a permanent monitoring site since 2003. It is located in a residential area and is influenced by traffic, with around 12,000 vehicles driving through the street every working day.^20^ The measuring Eiba site is located on the second floor of a regular apartment house. The site is part of the German Ultrafine Aerosol Network (GUAN^21,22^ ) the device inlets located on the northern side of the street, about 6 m above the street.

**Figure 1:**
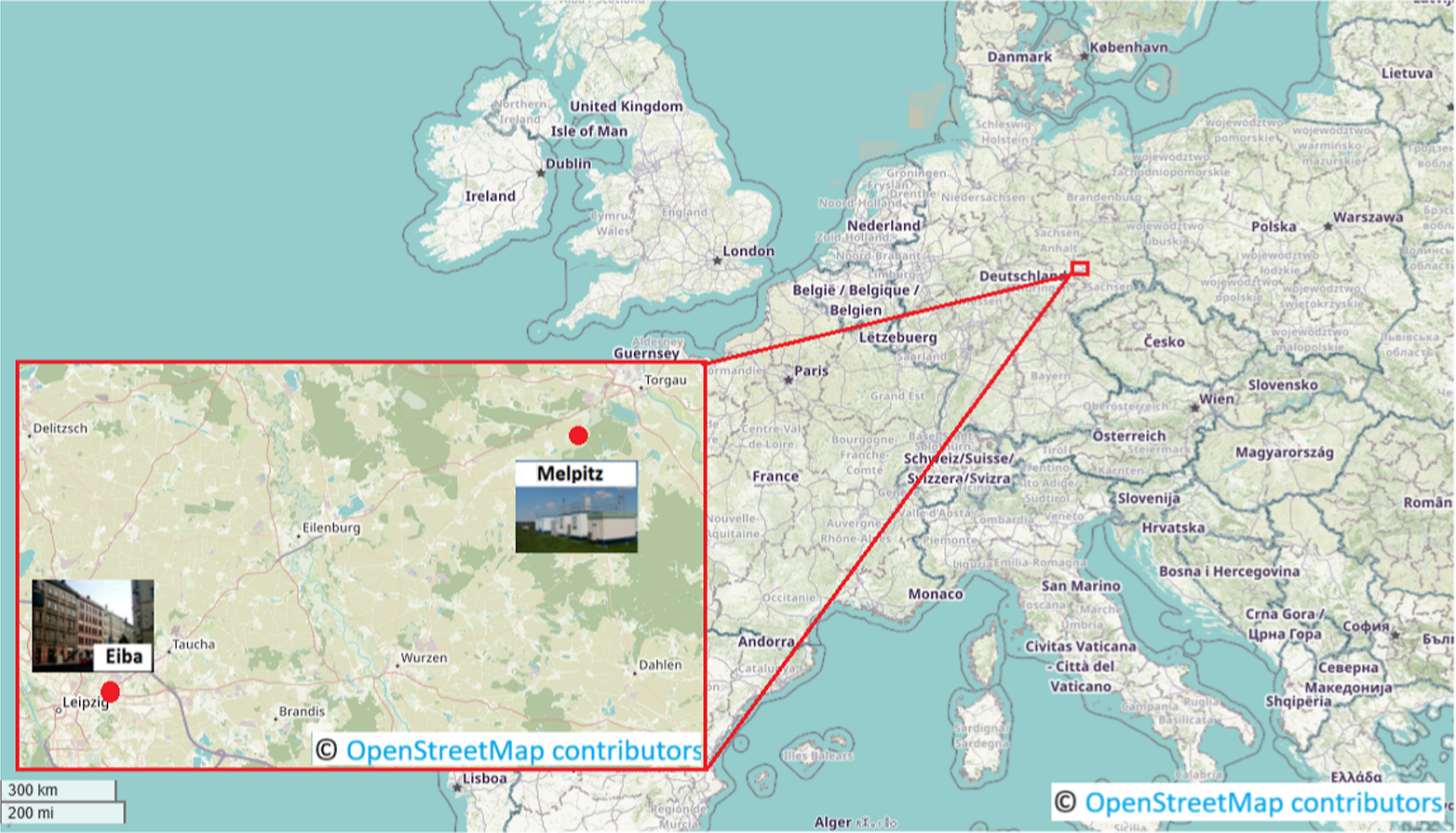
Geographical locations of the two measurement sites in eastern Germany. The urban site is in Leipzig (Eisenbahnstraβe, Eiba), and the rural background station is in Melpitz, ∼50 km northeast. Their alignment allows analysis of air mass transport under contrasting wind regimes. Depending on wind direction, Melpitz acts as upwind or downwind site. © OpenStreetMap contributors. Data available under the Open Database License (https://www.openstreetmap.org/copyright).

Melpitz research site (51°31′32″ N, 12°55′40″ E, 86 m a.s.l.) is situated in a meadow surrounded by agricultural land near Torgau, approximately 50 km northeast of Leipzig.^23^ This site, Melpitz, has operated since 1992 and is considered a rural background site.^24^ It is part of the European Research Infrastructure for the Observation of Aerosol, clouds, and trace gases (ACTRIS) network, the Measurement and Evaluation Program (EMEP), and it is one of the regional sites of the Global Atmosphere Watch (GAW).

### 2.2 Instrumentation

This work utilized two types of aerosol mass spectrometers during winter 2017, from January 25 to March 17. The High-Resolution Time-of-Flight Aerosol Mass Spectrometer (HR-ToF-AMS or simply AMS^25,26^ ) and the Aerosol Chemical Speciation Monitor (ACSM^16^) were employed. The ACSM has been continuously measuring at Melpitz since 2012, and a description of the setup and the measurements can be found in.^27–29^ The AMS was specially deployed for this project at the Eiba site. The ACSM and AMS utilize similar techniques for analyzing aerosol particles. The main difference remains in their respective time resolution (30 min for the ACSM and 5 min for the AMS). For the ACSM, QA/QC procedures, including mass reconstruction and evaluation of instrumental uncertainties, are described in detail in.^27^ Similarly, for the AMS, detailed methodologies can be found in.^30^

Because aerosol mass spectrometers can only detect the nonrefractory (NR) part of the particles, equivalent Black Carbon (eBC) was measured at both sites using a Multi-Angle Absorption Photometer (MAAP; model 5012, Thermo Scientific) within the PM_10_ range. To estimate the eBC mass concentration in the PM_1_ fraction, the eBC­(PM_10_) data was multiplied by a constant factor of 0.9, following the method described by^30^ and will be referred to in this study as eBC­(PM_1_). In addition to the MAAP, a dual Scanning Mobility Particle Sizer (TROPOS-type T-SMPS) at Melpitz and a Twin Differential Mobility Particle Sizer (TDMPS) at Eiba were used to measure the particle number size distribution (PNSD) from 3 to 800 nm for Melpitz and from 5 to 800 nm for Eiba (mobility diameter, *d*_mob_) at both ambient and 300 °C temperatures. All online instruments shared the same inlet and were sampled from the identical PM_10_ inlet postdryer, with an isokinetic splitter ensuring equitable distribution of sampled air among the instruments.^27^

In parallel to the online aerosol measurements, daily PM_2.5_ samples were collected at the two sites using a high-volume sampler (DIGITEL DHA-80, Digitel Elektronik AG, Hegnau, Switzerland) equipped with a quartz filter for Melpitz, and a low-volume sampler (Derenda Low Volume Sampler, PNS 16 T) at Eiba equipped with 47 mm quartz filters. Both samplers were collected from midnight to midnight. For detailed information about sample preparation and evaluation methods, see.^8,31^ Following the technique of^32^ levoglucosan, a tracer for wood-burning combustion, was measured using high-performance anion exchange chromatography coupled with an electrochemical detector (HPAEC-PAD) at Melpitz, and an ion-exchange chromatography system with pulsed amperometric detection (ICS3000, Dionex, USA) at Eiba. NO and NO_2_ levels at Melpitz were assessed using a customized Trace Level NO_x_ Analysis model 42i-TL (Thermo Scientific) equipped with a blue light converter, and at Eiba using Nitrogen Oxygen Analysers (model 8841, Monitor Laboratories). At the Melpitz site, NO and NO_2_ were combined as NO_x_ and used as an external variable in the PMF analysis (next section) to support factor identification. Additionally, routine measurements, including temperature, relative humidity, solar radiation, precipitation, wind direction, and wind speed, have been analyzed at both sites.

The QA/QC procedures, including mass and volume closure analyses, was applied to ensure the reliability and comparability of aerosol measurements across the two sites. A detailed description of these procedures, along with supporting comparisons to SMPS and filter-based measurements, is provided in the Supporting Information (Section 1).

### 2.3 PMF Analysis

The Positive Matrix Factorization (PMF) technique was employed to attribute the organic compounds measured by the AMS and ACSM to their respective sources. PMF was conducted on the combined AMS and/or ACSM data set using the Source Finder Pro tool (SoFi Pro, Datalystica Ltd., Villigen, Switzerland).^33^ This tool utilizes the multilinear engine (ME-2 ^34^) as a PMF solver. The PMF model aims to represent the original matrix *X*, which contains information on the concentration of each variable in time, as a product of the matrices *G* and *F*. In this context, *G* represents the contribution of source emission factors, and *F* represents the spectral “fingerprint” (spectrum) associated with each factor. As a result, a residual matrix *E* is generated. The PMF principle is expressed in eq 1

1 X=GF+E

In particular, the variables *F*_*kj*_ and *G*_*ik*_ are utilized to denote the time series and the chemical fingerprint of sources, respectively. The dimensions of *F*_*kj*_ and *G*_*ik*_ are determined by the order *p*, which represents the number of factors chosen to represent the user-defined data

2 $$X_{ij}=\sum_{k=1}^{p}G_{ik}\times F_{kj}+E_{ij}$$

In this study, two separate PMF inputs were prepared for measurements at the Eiba and Melpitz sites. PMF was conducted independently at each site, resulting in different factor identifications. The PMF analysis was performed using AMS data at a 5 min resolution, while for the ACSM, hourly PMF results from^29^ were used. The PMF analysis procedure for both sites is discussed in the Supporting Information file (Supporting Information 2).

### 2.4 Cross-Correlation

To assess the temporal relationship between two time series, such as PM_1_ concentrations measured at two monitoring sites, cross-correlation analysis was used. This method quantifies the similarity between two data sets as one is shifted in time relative to the other. It is particularly useful in identifying whether changes observed at one site follow those observed at another, which can suggest transport or shared influences. For each time shift (lag), a correlation coefficient is calculated using the following equation

3 $$r_{m}=\frac{\sum \left(x_{i}-\mathrm{x̅}\right)\left(y_{i-m}-\mathrm{y̅}\right)}{\sqrt{\sum {\left(x_{i}-\mathrm{x̅}\right)}^{2}\sum {\left(y_{i-m}-\mathrm{y̅}\right)}^{2}}}$$

Here *x*_*i*_ and *y*_*i*_ represent the values of the two time series, *x̅* and *y̅* are their respective means, and *m* is the lag in time steps (hours). The summation is performed only over the overlapping portions of the time series for each lag.^35^ Detailed information can be found at https://www.nhm.uio.no/english/research/resources/past/. In this study, the cross-correlation figure was obtained by the Past 4.17 software, which is available at https://www.nhm.uio.no/english/research/resources/past/.

To ensure consistent temporal resolution and minimize local variability in the cross-correlation analysis, all harmonization procedures, time lag considerations, and the impact of diurnal patterns were carefully addressed. A full description of the methodology—including temporal averaging, filtering criteria, treatment of time lags, and the removal of diurnal cycles—is provided in the Supporting Information (Section 3).

### 2.5 Urban Increment

In order to compare the two sites, the Urban Increment (UI) was determined using the widely recognized Lenschow’s approach.^19^ This method is typically employed to estimate the local urban contribution to atmospheric pollutant levels by comparing concentration data from two stations—commonly an urban site and a nearby suburban or rural background site. The key assumption behind this approach is that both sites are influenced by the same air mass under near-identical meteorological conditions, with minimal additional emissions occurring between the two locations. While this method has traditionally been applied to low time-resolution filter-based measurements (e.g.,^36–39^ among others), its use in this study with high time-resolution (hourly) online aerosol mass spectrometry data presents a distinct advantage. The higher resolution allows for more precise detection of concentration differences between the two sites, as well as for accounting for the transport time lag between them.

To address this, a cross-correlation analysis was performed between the two data sets to identify the optimal time lag, corresponding to the average transport time of air masses from Melpitz (background) to Eiba (urban). This temporal alignment enables a more accurate and representative estimation of the urban increment by ensuring that the compared data points correspond to the same air mass at different points along its trajectory. Within the present study, the UI is calculated by subtracting the hourly average values of Melpitz from those of Eiba using the following equation

4 $$\left[urban\;increment\right]=\left[{urban}_{Eiba}\right]-\left[{rural\text{\_}background}_{Mel}\right]$$

## 3 Results and Discussion

### 3.1 Chemical Composition of PM_1_

Figure 2 illustrates the temporal variations in mass concentration and the contributions of PM_1_ chemical components, alongside key meteorological parameters, recorded at the Melpitz (Figure 2a) and Eiba (Figure 2b) sites during the study period from January 25, 2017, to March 17, 2017. At both sites, the temperature displayed distinct diurnal cycles, ranging from near-freezing conditions at night to warmer temperatures during the day.

**Figure 2:**
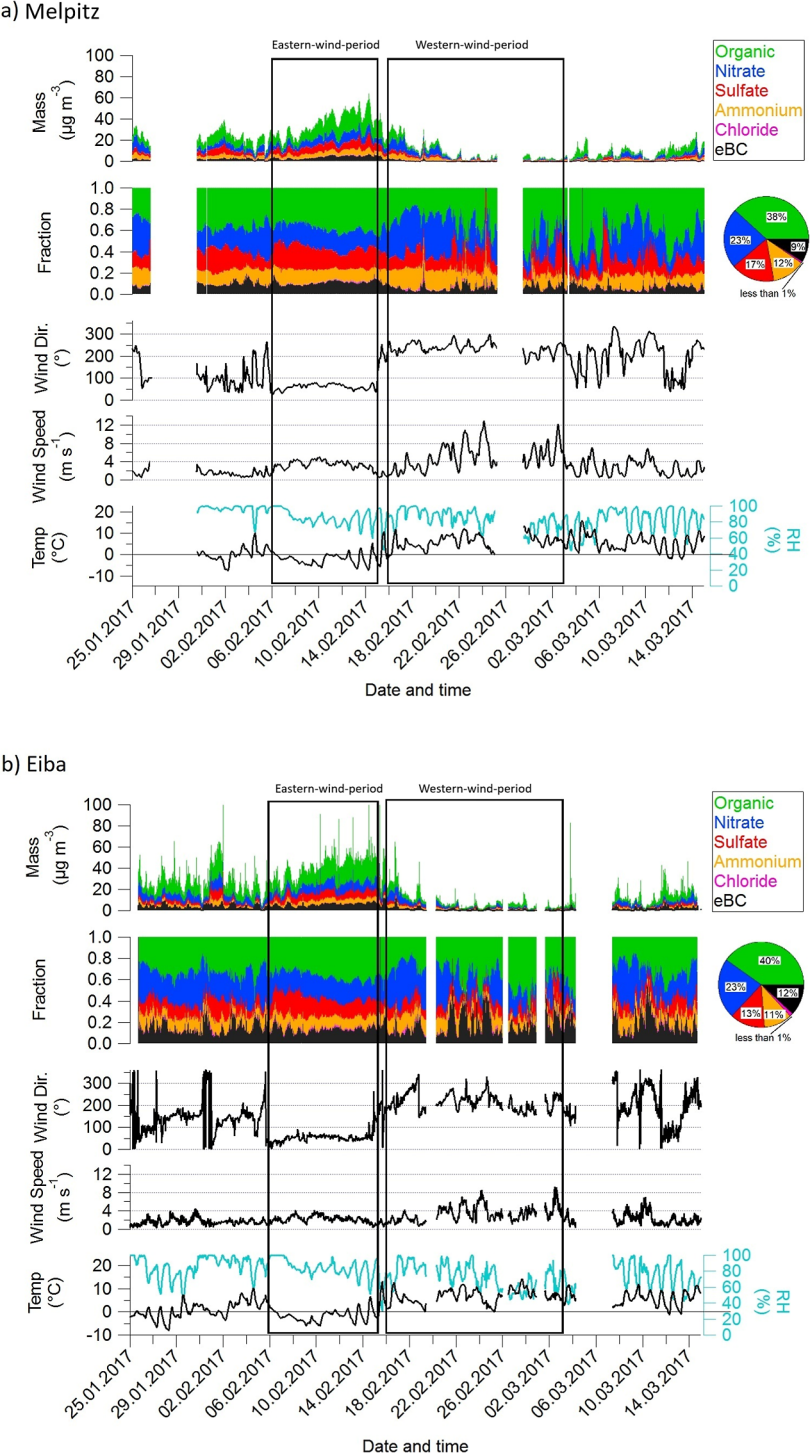
Time series of PM_1_ chemical composition and meteorological parameters at (a) Melpitz and (b) Eiba during winter 2017 (time in UTC). Panels display PM_1_ species mass, species fractions, and meteorological data. “Eastern-wind-period” and “Western-wind-period” denote distinct air mass regimes based on wind direction. Pie charts summarize average PM_1_ composition at each site for the entire measurement period.

Since Melpitz is a rural site, whereas Eiba is an urban site, this distinction plays a significant role in understanding the differences in aerosol properties and dynamics between the two sites. Both sites exhibited episodic variations in aerosol mass concentration. Eiba exhibited distinct peaks associated with local emissions from traffic and residential heating, which the AMS captured with higher temporal resolution. The mean PM_1_ mass concentration was lower at Melpitz (8.33 μg m^–3^) than at Eiba (17.51 μg m^–3^) (Figures 2 and S8a). Despite these short-term fluctuations, the overall trends in PM_1_ and chemical composition at both sites remained similar (Figure 2).

The wind direction and wind speed measurements at both Melpitz and Eiba were highly consistent and remarkably stable during two distinct periods, indicating the dominance of stable regionally representative air mass transport over several days. These periods corresponded to a persistent easterly wind (ranging from 35° to 140°) from February 6 to February 14, 2017, and a westerly wind (ranging from 210° to 320°) from February 16 to March 2, 2017. To ensure that these episodes reflected regional circulation patterns rather than local variability, it was essential for both sites to exhibit the same prevailing wind direction simultaneously, which was indeed the case during these intervals. Consequently, these periods were selected and clearly marked in the figures with black boxes labeled “Eastern-wind-period” and “Western-wind-period”. Given the geographical alignment of the two stations (refer to Figure 1), it can be reasonably assumed that a direct atmospheric connection exists during these specific wind regimes. During the Eastern-wind-period, Melpitz was located upwind of Eiba, allowing regional air masses to flow from the rural site toward the urban area. Conversely, during the Western-wind-period, Melpitz was downwind of Leipzig, resulting in a greater influence from urban outflow.

Furthermore, the meteorological conditions varied significantly between the two periods. The Eastern-wind-period was marked by lower temperatures (both stations −2 °C on average) and weaker wind speeds (both stations 3 m s^–1^ on average), characteristic of stable continental air masses. The Western-wind-period experienced relatively warmer temperatures compared to the Eastern-wind-period (both stations 6 °C on average) and stronger winds (both stations 5 m s^–1^ on average), consistent with maritime air masses. These contrasting meteorological conditions help clarify the differences in aerosol concentrations and chemical composition observed during each period. During the Eastern-wind-period, both sites exhibited higher total PM_1_ mass concentrations compared to the Western-wind-period, with values of 35.50 μg m^–3^ at Melpitz and 37.47 μg m^–3^ at Eiba (Figures 2 and S8). In contrast, during the Western-wind-period, the air masses were influenced by cleaner maritime air from the Atlantic Ocean,^29^ resulting in significantly lower PM_1_ concentrations at both sites. This Western-wind-period air mass resulted in lower aerosol mass concentrations than the Eastern-wind-period air mass at both sites, 8.31 and 6.93 μg m^–3^, Melpitz and Eiba, respectively. This wind dependency agrees with previous works at the two sites (e.g.,^28,29,31,36^ ). A detailed comparison will be discussed in the following section.

To further evaluate the role of meteorological factors in shaping observed aerosol concentrations, a short case study period (31 January to 1 February 2017) was examined, referred to as Case.S. This period exhibited meteorological conditions (e.g., wind speed and temperature) comparable to the Western-wind-period but featured predominantly easterly winds. PM_1_ concentrations during Case.S were elevated compared to the Western-wind-period period but remained below the levels observed during the full Eastern-wind-period. These findings support the conclusion that air mass origin plays a primary role in shaping aerosol loadings at both sites, even under similar dispersion conditions. The results of this case study are provided in the Supporting Information (Section 4).

To better compare the time series of the two sites, the total PM_1_ mass and chemical species were directly compared in Figure S8. In summary, the total PM_1_ mass concentration measured at Melpitz closely mirrored the levels recorded at Eiba (Figure S8a). There were only a few instances where PM_1_ concentrations at Eiba (indicated by the blue in Figure S8a) dropped below those at Melpitz. Additionally, there were short periods when PM_1_ levels at Eiba were higher than at Melpitz (marked by the red areas in Figure S8a). The similarity between the two sites indicates that a comparable background of aerosols influenced them. This is supported by the cross-correlation analysis conducted during the two periods (Figure 3), which shows a strong correlation (∼0.9 for total PM_1_) at both wind regimes, indicating consistent transport and emissions from upwind and downwind sources that influence both monitoring locations.

**Figure 3:**
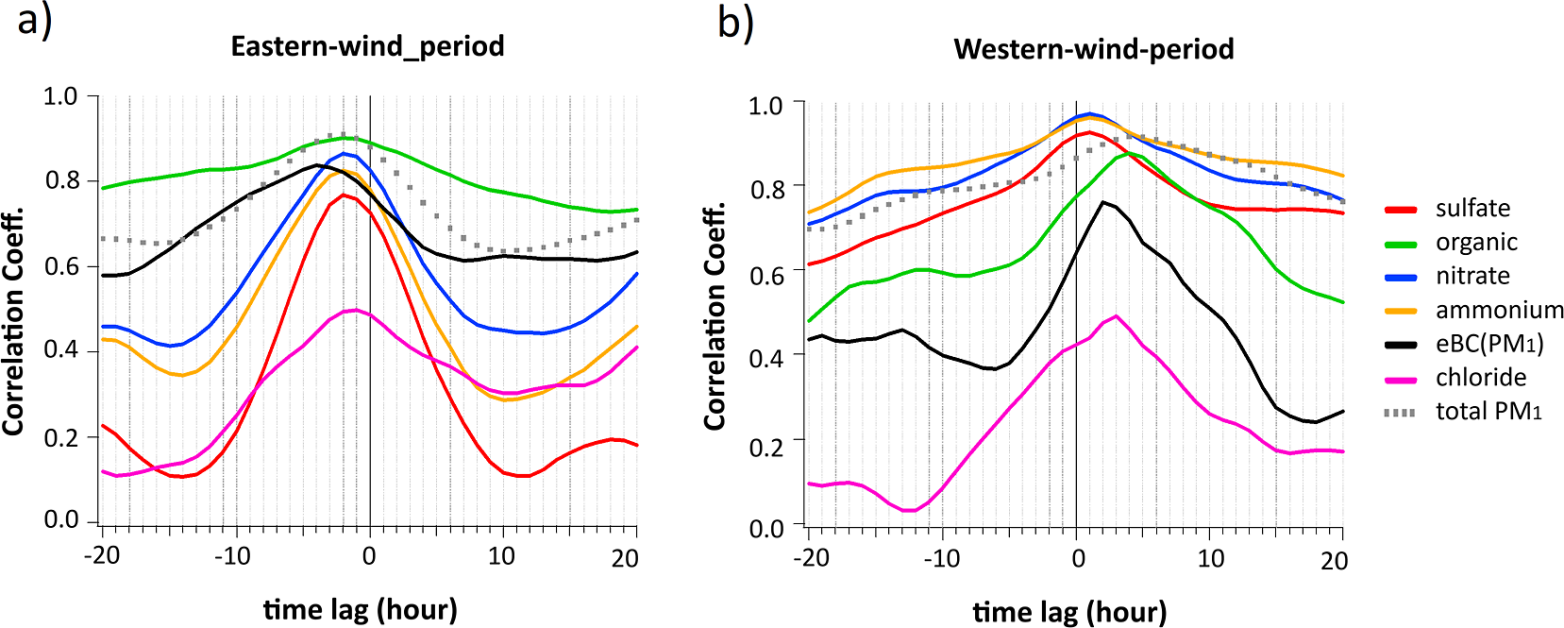
Cross-correlation coefficients between Melpitz and Eiba for PM_1_ chemical species during (a) Eastern and (b) Western wind-periods. The analysis was performed using time lags from −20 to +20 h, with a positive lag indicating that the Eiba data set was shifted forward relative to Melpitz.

A systematic comparison between the two sites for each species will be made in the following section.

#### 3.1.1 Organic Aerosol (OA)

Throughout the entire study period, OA consistently comprised the majority of the aerosol composition at both the Melpitz and Eiba sites, accounting for 38% and 40% of the total PM_1_, respectively (see Figure 2). However, the absolute concentrations of OA exhibited significant temporal variations (refer to Figures 2 and S8b). The mean OA mass concentration at Eiba (6.43 μg m^–3^) was higher than at Melpitz (3.36 μg m^–3^) during the observation period. This difference reflects the impact of local anthropogenic sources, notably traffic emissions. Furthermore, during the Eastern-wind-period, both sites recorded elevated mean mass concentrations of OA—13.83 μg m^–3^ at Melpitz and 14.35 μg m^–3^ at Eiba—compared to the Western-wind-period, 2.58 μg m^–3^ and 2.06 μg m^–3^ for Melpitz and Eiba, respectively (see Figures 2 and S8).

The cross-correlation analysis (Figure 3) shows that OA exhibited a strong correlation during the Eastern-wind-period, peaking slightly above 0.8. This correlation featured a time shift of approximately −2 h (h) to Eiba, indicating that OA concentrations at Melpitz tended to precede those at Eiba. This observation aligns with an estimated wind speed of 5 m s^–1^ recorded at both sites (Figure 2). Additionally, the geographical proximity of the two sites on a flat plain without significant relief further supports this time lag in OA detection. This strong correlation highlights the regional contribution of OA during the Eastern-wind-period, suggesting that both sites experienced similar regional-scale atmospheric processes. The broad correlation peak suggests that while regional dynamics influenced OA concentrations, the impact from local sources was minimal at both sites during this period. This further emphasizes the importance of understanding how regional atmospheric conditions can govern aerosol properties and dynamics across different environments, whether urban or rural. In contrast to the Eastern-wind-period, during the West wind period, OA maintained a high correlation (∼0.8) but with a time shift of +4 h to Melpitz and a sharper correlation peak, supporting the fact that it is downwind of the city of Leipzig. This indicates that OA’s urban sources and transport pathways might be at play.

#### 3.1.2 Nitrate

Following OA, nitrate represented a 23% contribution to total PM_1_ at both sites (Figure 2); however, the nitrate mean mass concentration was higher in Eiba (4.35 μg m^–3^) than in Melpitz (2.03 μg m^–3^) (Figure S8), likely due to the NO_x_ emissions contributing to nitrate formation at the Eiba site. Nitrate levels increased during colder periods, as seen during the Eastern-wind-period (6.55 μg m^–3^ and 7.84 μg m^–3^ at Melpitz and Eiba, respectively). This is consistent with the temperature-dependent gas-to-particle partitioning of ammonium nitrate, which favors the particle phase under lower temperatures as well as NO_3_ radical chemistry-driven nitrate formation. The trend was more pronounced at Eiba, where colder temperatures and higher relative humidity (RH) during Eastern-wind periods facilitated nitrate formation. In contrast, nitrate concentration decreased during the Western-wind-period period, with nearly the same concentrations for both Melpitz and Eiba (2.72 and 2.23 μg m^–3^, respectively), due to the cleaner air mass and reduced precursor availability. This pattern is consistent with findings from,^36^ who also reported higher nitrate concentrations during Eastern-wind-period conditions at these sites.

Regarding the cross-correlation (Figure 3), nitrate exhibited a strong peak (above 0.8) during the Eastern-wind-period, with the same time lag as OA (near *t* = −2 h). However, its peak shape clearly differed from the OA, suggesting some local nitrate formation at Eiba, likely related to traffic emissions. During the Western-wind-period, nitrate exhibited an even stronger correlation (close to 1) with a broad peak around *t* = +1 h. The slightly higher correlation under Western-wind-period suggests that nitrate production and transport were more consistent in this wind direction, potentially driven by specific regional sources or stable atmospheric conditions. This highlights the crucial role of wind patterns in shaping nitrate concentrations and their relationship with other pollutants in urban environments. Understanding these dynamics is essential for developing effective air quality management strategies.

#### 3.1.3 Sulfate

Sulfate contributed more to PM_1_ at Melpitz (17%) than Eiba (13%; Figure 2). This higher level of sulfate mass contribution can be explained by the role of solid fuels, such as wood, biomass, and coal, for residential heating usage and oxidation processes (e.g., SO_2_ oxidation).^29^ The combustion of these sulfur-containing compounds results in increased concentrations and the contribution of sulfate emissions to PM_1_ (Figure S8). However, during the Eastern-wind-period, sulfate levels increased at both sites (6.79 μg m^–3^ and 19%, 6.12 μg m^–3^ and 16%; at Melpitz and Eiba, respectively), driven by transport of aged aerosols from Eastern Europe. In contrast, sulfate concentrations decreased during the Western-wind-period (1.10 μg m^–3^ and 13%, 0.71 μg m^–3^ and 10% at Melpitz and Eiba, respectively), reflecting the influence of maritime air masses with lower sulfate precursor concentrations. This trend is consistent with,^36^ who also reported higher sulfate concentrations during Eastern-wind-period at both sites.

During the Eastern-wind-period, sulfate showed a peak correlation centered near *t* = −2 h, with values exceeding 0.7, indicating its strong regional influence and well-mixed nature under Easterly winds. However, during the Western-wind-period, sulfate showed a broad and strong correlation (∼0.9), centered at *t* = +1 h. This consistent pattern reflected sulfate’s regional dominance and long atmospheric lifetime, with little dependence on wind direction. The broad peak during the Western-wind-period reflected the secondary nature of sulfate, as it was primarily formed through atmospheric oxidation processes (e.g., SO_2_ oxidation) over larger spatial scales. This secondary formation process, combined with the long atmospheric lifetime of sulfate, allowed it to be transported regionally, resulting in synchronized variations between the sites.

#### 3.1.4 Ammonium

Ammonium mass contributions were similar at both the Melpitz and Eiba sites, accounting for 12% and 11% of the total PM_1_, respectively (see Figure 2). Despite the comparable concentrations of ammonium at both locations—2.03 μg m^–3^ at Eiba and 1.10 μg m^–3^ at Melpitz—it plays a critical role as a neutralizing agent for nitrate and sulfate. At Eiba, the presence of ammonium was primarily linked to urban activities, whereas at Melpitz, it indicated regional transport of secondary inorganic aerosols. Ammonium levels increased significantly during the Eastern-wind-period, reaching 4.22 μg m^–3^ at Eiba and 4.15 μg m^–3^ at Melpitz, driven by the accumulation of ammonium nitrate and ammonium sulfate. Conversely, during the West-wind-period, ammonium concentrations declined to 0.92 μg m^–3^ at Eiba and 1.33 μg m^–3^ at Melpitz, mirroring changes in nitrate and sulfate levels.^36^ reported similar changes, with higher ammonium concentrations in the Eastern-wind-period and lower concentrations in the Western-wind-period for both locations.

Based on the cross-correlation plot, ammonium demonstrated a high correlation, peaking at approximately 0.8 during the Eastern-wind-period and above 0.9 in the Western-wind-period. This correlation pattern was similar to that observed for sulfate and nitrate, highlighting ammonium’s role as a neutralizing agent for these acidic species. The broad peak in the correlation suggests that ammonium was regionally distributed, particularly under Eastern-wind-period, which was likely influenced by its relationship with secondary aerosol formation processes.

#### 3.1.5 Chloride

Chloride levels were consistently higher at Eiba (0.22 μg m^–3^) compared to Melpitz (0.05 μg m^–3^), reflecting dominant urban sources such as coal and wood combustion, domestic heating, and occasional waste incineration.^40^ A minor contribution from wintertime road salting may also be present. These short-lived and spatially confined events, particularly under Eastern-wind-period, led to sharp chloride peaks at Eiba that were not observed at Melpitz. Importantly, these chloride peaks at Eiba temporally align with CCOA (coal combustion OA; CCOA will be discussed further in the next sections) enhancements (Figure S9), suggesting a common source origin. However, it is important to note that AMS and/or ACSM technology have limitations in detecting salt.^41^ The primary reason is that most chloride resides in the refractory portion, which cannot be flash vaporized at 600 °C. As a result, the chloride identified by AMS and/or ACSM is predominantly associated with combustion processes including wood and coal burning, as well as trash incineration.^40^ Furthermore, AMS generally offers lower detection limits for chloride than the ACSM, which means it can detect smaller chloride concentrations in aerosol particles. Therefore, chloride demonstrated the lowest correlation among all components in the cross-correlation plot, with a weaker peak of around 0.4 for both wind directions.

#### 3.1.6 eBC­(PM_1_)

eBC­(PM_1_) displayed lower mass contributions and concentrations at Melpitz compared to Eiba, with values of 9% and 1.55 μg m^–3^ for Melpitz, versus 12% and 2.15 μg m^–3^ for Eiba (see Figures 2 and S8). The elevated eBC­(PM_1_) levels at Eiba indicate emissions linked to combustion sources, typical of urban areas, particularly traffic. However, comparing the two time series (Figure S8) indicates that the elevated eBC­(PM_1_) average mass concentration was primarily due to short, high peaks at Eiba. Temporal alignment between eBC­(PM_1_) and OA factors (such hydrocarbon-like OA (HOA), biomass-burning OA (BBOA), coal combustion OA (CCOA); the factors which will be discussed in next sections) further supports the attribution of these peaks to primary combustion sources, particularly during cold-season urban emissions (Figure S9). Between these peaks, the concentration reaches levels that are quite similar to those measured at Melpitz. This trend is particularly evident during the Eastern-wind-period. Both monitoring sites recorded peak eBC­(PM_1_) levels during the Eastern-wind-period, reaching 3.88 μg m^–3^ at Melpitz and 4.43 μg m^–3^ at Eiba, which could also be linked to LRT emissions. Notably, eBC­(PM_1_) concentrations diminished significantly during the Western-wind-period, dropping to 0.48 μg m^–3^ at Melpitz and 0.90 μg m^–3^ at Eiba. This decrease is attributed to the influx of cleaner air masses affecting both locations.

Based on cross-correlation, eBC­(PM_1_) exhibited a strong correlation (∼0.8) centered at *t* = −4 h, indicating a significant regional component to eBC­(PM_1_) emissions under Eastern-wind-period. Despite being a primary pollutant, the strong correlation implied consistent transport and emissions from upwind sources influencing both monitoring locations. Under the Western-wind-period, eBC­(PM_1_) showed a moderate correlation (∼0.7). The reduced correlation suggested that localized eBC­(PM_1_) sources, such as emissions or industrial activities from Leipzig city, played a more prominent role under this wind regime.

### 3.2 OA Sources of PM_1_

The comparison between the two sites showed the importance of the background aerosol to the urban aerosol chemical composition. To investigate the influence of different anthropogenic sources, source apportionment analysis of the OA was performed separately for Melpitz and Eiba using the PMF model. Detailed steps for factor identification at both sites are provided in the Supporting Information 2. The analysis revealed site-specific differences in the factors. The selected OA source apportionment solution, however, identified five distinct factors based on their time series and mass spectra for both sites (Figures 4 and S4). These factors were classified as hydrocarbon-like OA (HOA), biomass-burning OA (BBOA), coal combustion OA (CCOA), and two types of Oxygenated OA: Less Oxidized OOA (LO-OOA) and More Oxidized OOA (MO-OOA).

**Figure 4:**
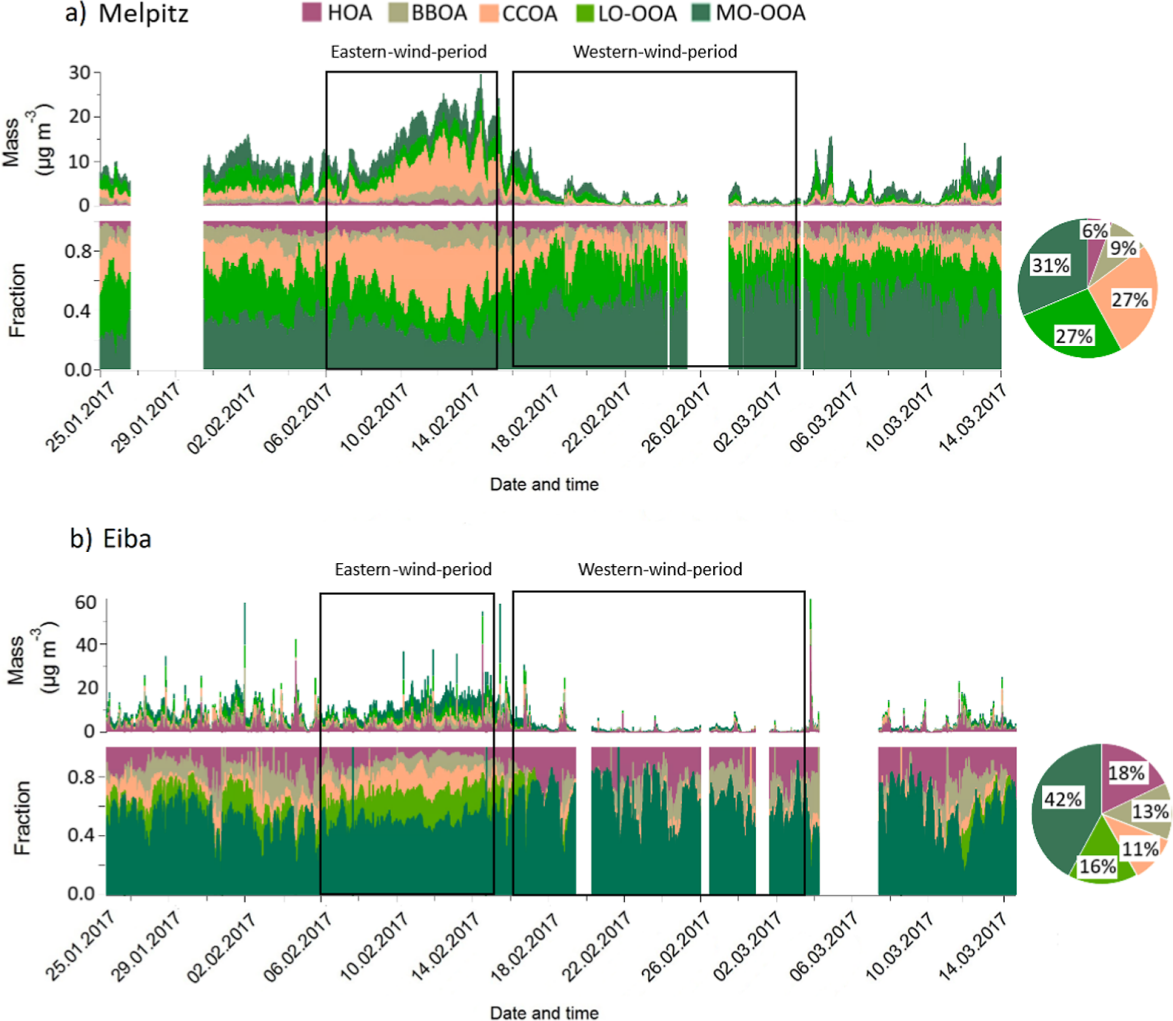
Time series of PMF-resolved OA sources at (a) Melpitz and (b) Eiba during winter 2017 (time in UTC). Panels display PMF factors mass, and fractions. “Eastern-wind-period” and “Western-wind-period” denote distinct air mass regimes based on wind direction. Pie charts summarize average PMF factors at each site for the entire measurement period.

#### 3.2.1 HOA

Comprehensive details on OA factors for Melpitz are available in previous studies of.^28,29^ At both sites, the Hydrocarbon-like OA (HOA) is characterized by specific peaks at *m*/*z* 41 (C_3_H_5_^+^), 43 (C_3_H_7_^+^), 55 (C_4_H_7_^+^), and 57 (C_4_H_9_^+^) associated with alkyl and alkenyl fragments^42^ (Figure S4). It is important to note that HOA is linked to traffic emissions and domestic heating fuels.^43^ Throughout the entire study period, including both the Eastern and Western wind-periods, the HOA concentration was notably higher at Eiba than at Melpitz. The mean mass concentration at Eiba over the entire time was 0.96 μg m^–3^, contributing to 18% of the OA, while at Melpitz, the mean was 0.41 μg m^–3^, accounting for just 6% of the OA (see Figures 4 and S10). Not surprisingly, the elevated levels of HOA at Eiba indicated ongoing local traffic emissions, as the site is situated in a street canyon. Additionally, the diurnal profile of HOA at Eiba (Figure S14b) shows peaks corresponding to the increased concentrations associated with traffic activity. In contrast, lower HOA levels at Melpitz indicated a greater reliance on liquid fuels for household heating in the region.^29,44^

From the cross-correlation plot during the Eastern-wind-period (Figure 5a), HOA showed low and inconsistent correlations across all time lags, even dipping into negative values. This lack of correlation confirmed that HOA was primarily emitted from highly localized sources, such as intense traffic at Eiba and/or lower domestic emissions at Melpitz, as previously mentioned. Furthermore, the negative correlation suggested that the emissions at the two sites were not happening simultaneously or consistently across the same timeframes due to the differences between source emissions. A better but still relatively low cross-correlation coefficient was obtained for the Western-wind-period (Figure 5b). It supports its localized emissions near urban and street areas with limited transport between the two sites, even under Western-wind-period. There was a slight increase in correlation at positive lags, which could be attributed to some downwind transport of emissions from Eiba to Melpitz.

**Figure 5:**
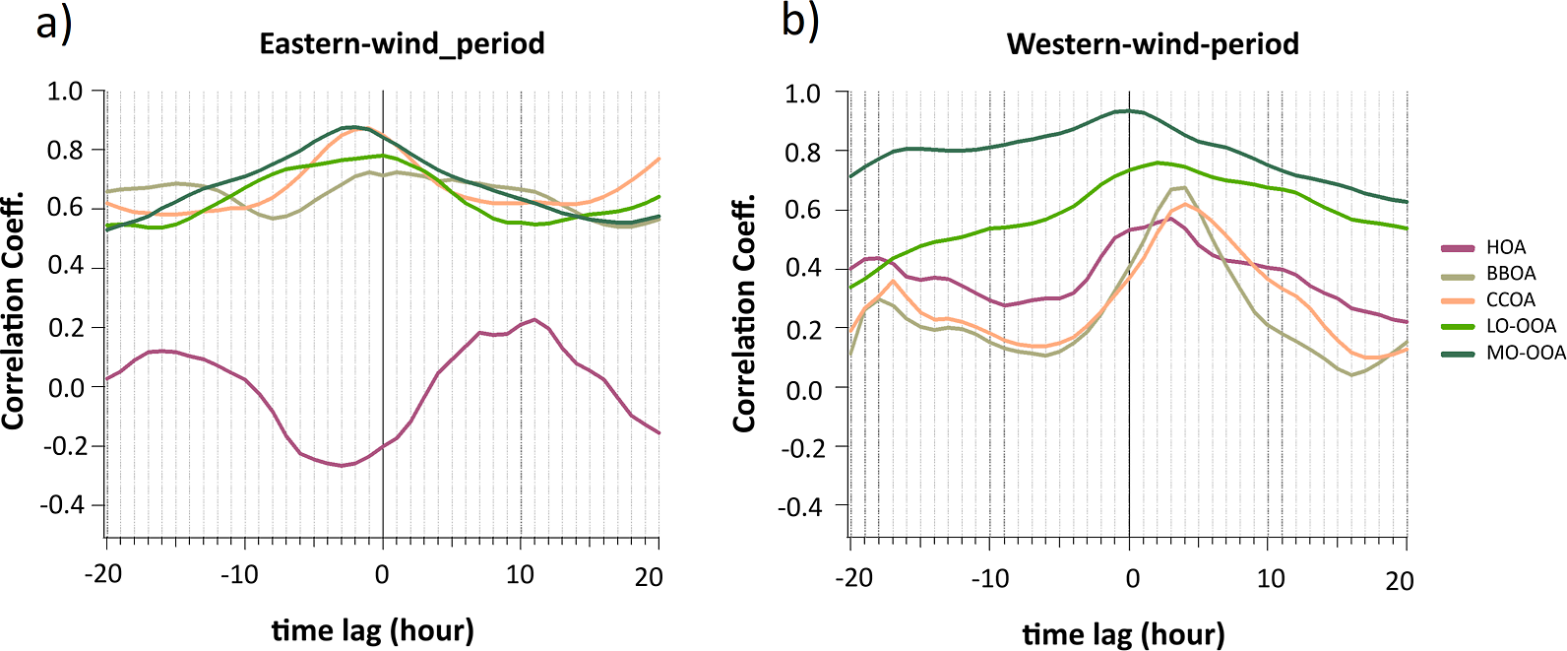
Cross-correlation coefficients between Melpitz and Eiba for PMF factors during (a) Eastern and (b) Western wind-periods. The analysis was performed using time lags from −20 to +20 h, with a positive lag indicating that the Eiba data set was shifted forward relative to Melpitz.

#### 3.2.2 BBOA

Biomass burning OA (BBOA) was distinguished by the significant presence of ions at *m*/*z* 60 (C_2_H_4_O_2_^+^) and 73 (C_3_H_5_O_2_^+^), which are fragments commonly associated with anhydrosugars like levoglucosan, a well-known marker of biomass burning emissions.^45,46^ BBOA was a significant contributor at both measurement sites, with mean mass concentrations of 0.70 μg m^–3^ (13% of OA) at Eiba and 0.67 μg m^–3^ (9% of OA) at Melpitz. This contribution was particularly notable during colder periods, reflecting the use of biomass burning for heating. During the Eastern-wind-period, both sites reached their maximum concentrations, with Melpitz reporting higher levels (1.61 μg m^–3^) than Eiba (1.23 μg m^–3^). This increase can be attributed to increased heating activity due to lower temperatures, as well as transported emissions from Eastern countries. The higher BBOA concentrations at Melpitz, as shown in Figure S10, can be linked to a stronger reliance on wood for heating in rural areas, in addition to emissions transported from the northeastern region near the site (see Figure S13). However, during the Western-wind-period, BBOA concentrations significantly decreased at both sites (Melpitz: 0.24 μg m^–3^, Eiba: 0.18 μg m^–3^), suggesting the influx of cleaner air masses from the West and reduced heating activities due to warmer temperatures.

The cross-correlation plot (Figure 5) indicates that BBOA exhibited a moderate correlation coefficient (∼0.7), peaking around lag zero during the Eastern-wind-period. This modest correlation suggests that biomass burning emissions were somewhat regional but had limited spatial dispersion, likely due to specific localized burning activities. Additionally, BBOA showed a moderate correlation with delayed peaks at positive lags during the Western-wind-period. This observation implies that biomass-burning emissions were transported from Eiba to Melpitz under westerly winds after a certain delay (approximately 4 h).

#### 3.2.3 CCOA

Coal combustion OA (CCOA) mass spectra are characterized by distinct fragments at *m*/*z* 77 (C_6_H_5_^+^), 91 (C_7_H_7_^+^), and 115 (C_9_H_7_^+^), which are associated with polycyclic aromatic hydrocarbons.^47^ CCOA, which stands for coal combustion emissions, is mainly associated with coal burning for energy production in power plants. Additionally, coal is a common source of domestic heating during the colder months. Compared to the previous anthropogenic factors (HOA and BBOA), the two CCOA mass spectra exhibit significant differences, especially regarding the presence of higher *m*/*z* 44 in the mass spectra obtained from Melpitz compared to those from Eiba. Therefore, the CCOA factor identified at Melpitz can be classified as a processed CCOA factor, which can be referred to as oxygenated-CCOA (OCCOA) with higher *m*/*z* 44, as reported by.^48^ In contrast, the CCOA from Eiba, with its lower contribution of *m*/*z* 44, is likely to be more freshly emitted. However, the mean mass concentration and contribution of OCCOA at Melpitz were significantly higher over the entire period, measuring 2.01 μg m^–3^, which accounts for 27% of OA. In contrast, Eiba showed lower levels, with a mean mass concentration of 0.58 μg m^–3^, contributing only 11% of OA. The reduced presence of CCOA in Eiba can be attributed to the prevalence of natural gas or centralized heating systems in urban areas, which diminishes the use of coal for heating. However, the Eiba measurement site was located on the street with restaurants that also use coal for cooking, which explains the presence of fresh CCOA in that area. Moreover, during the Eastern-wind-period, OCCOA showed the highest mass concentration in Melpitz (6.08 μg m^–3^) (Figure S10), which can be due to the LRT from Eastern European countries. In contrast, Eiba showed only 1.41 μg m^–3^ of CCOA mass concentration during the Eastern-wind-period, which could be linked to the fresh CCOA emissions, mainly from the restaurants around. The exact explanation for the difference in CCOA splitting between the two sites remains unclear, particularly regarding how the OCCOA from Melpitz affects the factor results at Eiba. At this stage, there is a strong suspicion that part of the OCCOA present at Eiba is incorporated into the more oxidized oxygenated organic aerosol (MO-OOA) factors. This is evident as the MO-OOA from Eiba is less oxidized than that from Melpitz. The MO-OOA from Melpitz is influenced by a mix of long-range transported MO-OOA and localized anthropogenic aging.

From the cross-correlation plot (Figure 5), CCOA demonstrated a moderately broad peak around the time lag zero during the Eastern-wind-period. Cooking-related emissions using coal were generally localized to Eiba areas. Still, its broader peak suggested that similar emission patterns at both sites may occurred simultaneously, likely due to shared urban cooking activity trends during specific times of the day. Similar to the Eastern-wind-period, a correlation remained moderate in the Western-wind-period, suggesting coal combustion emissions preliminary influenced local areas.

#### 3.2.4 LO-OOA

The two less oxidized oxygenated OA (LO-OOA) showed variability at *m*/*z* 43 (C_2_H_3_O^+^ and C_3_H_7_^+^) and 44 (CO_2_^+^) and were influenced by other ions, such as *m*/*z* 29 (CHO^+^ or C_2_H_5_^+^) and 55 (C_4_H_7_^+^), which aligns with findings from other studies.^29,47,49^ LO-OOA exhibited a mean mass concentration of 0.86 μg m^–3^ (16% of total OA) at Eiba and 1.97 μg m^–3^ (27% of total OA) at Melpitz. Melpitz consistently recorded higher LO-OOA concentrations than Eiba (Figure S10), which was attributed to the regional transport of precursors. This phenomenon was particularly evident during the Eastern-wind-period, with mean mass concentrations of 2.13 μg m^–3^ and 3.21 μg m^–3^ at Eiba and Melpitz, respectively. However, LO-OOA levels decreased slightly during Western-wind-period transport compared to Eastern-wind-period. Despite this decrease, Melpitz still recorded higher concentrations than Eiba (0.88 and 0.12 μg m^–3^, respectively). This reflects reduced regional precursor transport under Western-wind-period flow conditions.

In terms of cross-correlation (Figure 5), LO-OOA exhibited a high and broad peak correlation near zero during the Eastern-wind-period, indicating minimal difference between the two sites. This broad peak suggests that LO-OOA was influenced by a mix of local and moderately transported regional sources. During the Western-wind-period, LO-OOA exhibited a lower correlation than during the Eastern-wind-period, suggesting that fresher SOAs were more effectively transported between sites under Western-wind-period. Still, the transport involved a slight delay due to the time taken for chemical reactions to occur and for the aerosols to reach the Melpitz. Compared to the Eastern-wind-period, this peak may also indicate stronger wind (Figure 2) and/or more consistent transport pathways under Western-wind-periods.

#### 3.2.5 MO-OOA

More oxidized oxygenated OA (MO-OOA) was characterized by a strong peak at *m*/*z* 44, indicating a higher oxidation state. However, the MO-OOA from Eiba was less oxidized than that from Melpitz. The mean mass concentration of MO-OOA was 2.26 μg m^–3^ (42% of total OA) at Eiba and 2.32 μg m^–3^ (31% of total OA) at Melpitz. MO-OOA concentrations were also elevated during Eastern-wind-period, with mean mass concentrations of 4 μg m^–3^ and 4.82 μg m^–3^ at Melpitz and Eiba, respectively, reflecting the aged and oxidized nature of aerosols transported over long distances from the East. In contrast, MO-OOA concentrations were lower during Western-wind-period flows compared to the Eastern-wind-period (Melpitz with 1.09 μg m^–3^ and Eiba with 0.94 μg m^–3^). The decline at both sites suggested a reduction in the transport of highly aged aerosols from Western-wind-period regions.

Similar cross-correlation patterns for MO-OOA were observed during both Eastern and Western wind-periods (Figure 5), with a broad peak centered at zero time lag between the two sites. These patterns support its well-mixed and regional nature, as MO-OOA represented aged SOAs that were transported over long distances and were less influenced by localized emission variability. In other words, the high correlation highlighted that MO-OOA was dominated by regional-scale processes such as atmospheric oxidation and long-range transport, which were less dependent on wind direction and made it the most spatially uniform component.

### 3.3 Urban Increment (UI) of PM_1_ Chemicals

Figure 6 shows the results of this approach in the study, which utilized the hourly time resolution of the data set from both stations. The time series plot (Figure 6a) shows the UI for the Eastern-wind-period concerning the PM_1_ components at the Eiba site. After February 12, 2017, the UI became more negative due to meteorological conditions (higher temperature, lower RH, and lower wind speed, as shown in Figure 2) and dilution. Therefore, the following discussion will focus on the periods when the UI is predominantly positive with more stable meteorological conditions (stable wind speed, temperature, and RH; Figure 2), which can be seen on the left side of the time series in Figure 6a.

**Figure 6:**
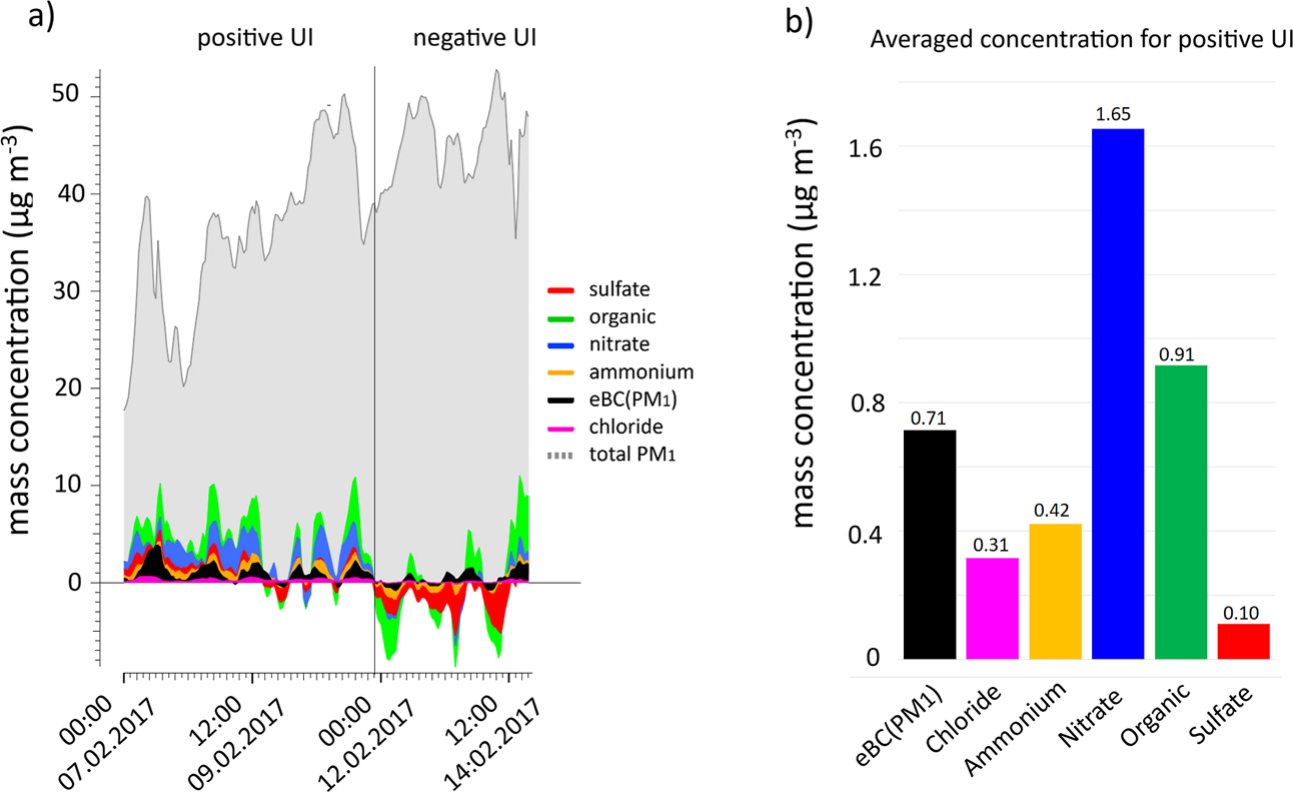
(a) Time series of PM_1_ components and estimated urban increment (UI) at Eiba during the Eastern-wind-period (7–14 February 2017). The stacked areas represent mass concentrations of sulfate, organic aerosol, ammonium, nitrate, equivalent black carbon (eBC­(PM_1_)), and chloride. The gray-shaded area and dashed line show total PM_1_ at Eiba station. Periods of positive and negative UI are indicated. (b) Averaged concentrations of PM_1_ components during positive UI.

The time series plot indicates that the urban contributions to the PM_1_ components at the Eiba site were relatively low (averaging 4.13 μg m^–3^; positive UI) during the Eastern-wind-period, accounting for about 9% of the total PM_1_ mass concentration. This suggests that the majority (91%) of PM_1_ originated from regional background sources transported into the city rather than from local urban emissions. Overall, the UI aerosol was dominated by nitrate (1.65 μg m^–3^, a secondary species), followed by organic (0.91 μg m^–3^), eBC­(PM_1_) (0.71 μg m^–3^), and chloride (0.31 μg m^–3^; Figure 6b). Distinct peaks in nitrate, eBC­(PM_1_), chloride, and OA suggest that these components can be associated with primary urban emissions such as traffic exhaust, house heating, and local SOA processes. The diurnal profiles (Figure S11) indicate that eBC (PM_1_) and OA peaked during the day, likely due to traffic and secondary aerosol formation in the city. Chloride follows a similar diurnal cycle, suggesting that its primary source in the city is industrial or urban activity. In contrast, sulfate levels remained low, averaging 0.10 μg m^–3^ (Figure 6b), indicating that sulfate was not primarily emitted within the city. Instead, it appears to be a secondary product mainly resulting from the oxidation of sulfur dioxide (SO_2_) emitted by nearby coal-fired home heating, power plants, and industrial activities located on the East side of the city. This suggests that sulfate formation is more influenced by regional atmospheric conditions rather than by immediate urban emissions.

The findings of this study highlight the importance of online techniques with high time resolution (AMS and ACSM) for analyzing aerosol transport and urban air quality in the PM_1_ size range for more accurate assessments. It also underscores the value of having sampling sites within the same air mass flow, which is advantageous for cross-correlation analysis. The results further emphasize the critical role of LRT in shaping urban air quality, supporting previous research by,^50,51^ which reinforces existing knowledge and contributes to ongoing studies in this field. However, we acknowledge that the short study period, like the current one focused on the winter season, limits the ability to generalize conclusions to an entire year with different seasons or other cities. Therefore, a more extensive, year-round study is needed to validate these findings and investigate seasonal variations. Given that current air quality regulations focus primarily on reducing emissions from major urban sources to meet the WHO standard of 5 μg m^–3^ for PM_2.5_,^3^ our findings suggest that the WHO regulation may be inadequate under meteorological conditions similar to those observed in the current study. Hence, a broader approach to air quality regulation, which considers rural areas and agricultural emissions on a larger scale—potentially across the entire European Union—should be explored.

## Supplementary Material



## Notes

The Supporting Information provides additional figures and methodological details that complement the main text. It includes instrument QA/QC procedures (Section 1, Figure S1), source apportionment of OA using PMF (Section 2, Figures S2–S4), and cross-correlation analysis (Section 3, Figures S5 and S6). Comparative time series of PM_1_ chemical composition between Melpitz and Eiba are shown in Figure S8, while Figure S9 presents eBC­(PM_1_) and chloride data alongside selected OA factors. Further comparisons of OA factors between the two sites are provided in Figure S10, with diurnal profiles during the UI period shown in Figure S11. Nonparametric wind regression results are discussed in Section 5 (Figures S12 and S13), and Figure S14 displays the diurnal variation of HOA at both locations (PDF)

## References

[1] World health statistics 2019: Monitoring health for the SDGs, sustainable development goals. World Health Organization: Geneva, 2019; License: CC BY-NC-SA 3.0 IGO.

[2] Nethery R.
C., Dominici F. (2019). Estimating
pollution-attributable mortality at the
regional and global scales: challenges in uncertainty estimation and
causal inference. Eur. Heart J..

[3] European Union . Directive - EU - 2024/2881 - EN - EUR-Lex. https://eur-lex.europa.eu/eli/dir/2024/2881/oj/eng (accessed 2025–02–14).

[4] Heal M. R., Kumar P., Harrison R. M. (2012). Particles, Air Quality, Policy and
Health. Chem. Soc. Rev..

[5] Shleag A. M., Shiha F. A., Aborba N. A. (2024). Environmental and Air Pollution’s
Impact on Health: Challenges and Opportunities. Int. J. Electr. Eng. and Sustain..

[6] Feng X., Xu X., Yao X., Zhao Y., Tang Y., Zhao Z., Wei Y., Mehmood T., Luo X. S. (2024). Sources, Compositions, Spatio-Temporal
Distributions, and Human Health Risks of Bioaerosols: A Review. Atmos. Res..

[7] Chen G., Canonaco F., Tobler A., Aas W., Alastuey A., Allan J., Atabakhsh S., Aurela M., Baltensperger U., Bougiatioti A., De Brito J. F., Ceburnis D., Chazeau B., Chebaicheb H., Daellenbach K. R., Ehn M., El Haddad I., Eleftheriadis K., Favez O., Flentje H., Font A., Fossum K., Freney E., Gini M., Green D. C., Heikkinen L., Herrmann H., Kalogridis A. C., Keernik H., Lhotka R., Lin C., Lunder C., Maasikmets M., Manousakas M. I., Marchand N., Marin C., Marmureanu L., Mihalopoulos N., Močnik G., Nęcki J., O’Dowd C., Ovadnevaite J., Peter T., Petit J. E., Pikridas M., Matthew
Platt S., Pokorná P., Poulain L., Priestman M., Riffault V., Rinaldi M., Różański K., Schwarz J., Sciare J., Simon L., Skiba A., Slowik J. G., Sosedova Y., Stavroulas I., Styszko K., Teinemaa E., Timonen H., Tremper A., Vasilescu J., Via M., Vodička P., Wiedensohler A., Zografou O., Cruz Minguillón M., Prévôt A. S. H. (2022). European Aerosol Phenomenology –
8: Harmonised Source Apportionment of Organic Aerosol Using 22 Year-Long
ACSM/AMS Datasets. Environ. Int..

[8] Velásquez-García M. P., Hernández K. S., Vergara-Correa J. A., Pope R. J., Gómez-Marín M., Rendón A. M. (2024). Long-Range Transport of Air Pollutants Increases the
Concentration of Hazardous Components of PM2.5 in Northern South America. Atmos. Chem. Phys..

[9] Casallas A., Cabrera A., Guevara-Luna M. A., Tompkins A., González Y., Aranda J., Belalcazar L. C., Mogollon-Sotelo C., Celis N., Lopez-Barrera E., Peña-Rincon C. A., Ferro C. (2024). Air Pollution Analysis
in Northwestern South America: A New Lagrangian
Framework. Sci. Total Environ..

[10] Jin X., Cai X., Huang Q., Wang X., Song Y., Kang L., Zhang H. (2022). PM2.5 Exchange
Between Atmospheric Boundary Layer and Free Troposphere
in North China Plain and Its Long-Range Transport Effects. J. Geophys. Res.:Atmos..

[11] van
Pinxteren D., Mothes F., Spindler G., Fomba K. W., Herrmann H. (2019). Trans-Boundary PM10: Quantifying Impact and Sources
during Winter 2016/17 in Eastern Germany. Atmos.
Environ..

[12] Daellenbach K. R., Uzu G., Jiang J., Cassagnes L. E., Leni Z., Vlachou A., Stefenelli G., Canonaco F., Weber S., Segers A., Kuenen J. J. P., Schaap M., Favez O., Albinet A., Aksoyoglu S., Dommen J., Baltensperger U., Geiser M., El Haddad I., Jaffrezo J. L., Prévôt A. S. H. (2020). Sources
of Particulate-Matter Air Pollution and Its Oxidative Potential in
Europe. Nature.

[13] Shi Y., Chen J., Hu D., Wang L., Yang X., Wang X. (2014). Airborne Submicron
Particulate (PM1) Pollution in Shanghai, China:
Chemical Variability, Formation/Dissociation of Associated Semi-Volatile
Components and the Impacts on Visibility. Sci.
Total Environ..

[14] Shrivastava M., Cappa C. D., Fan J., Goldstein A. H., Guenther A. B., Jimenez J. L., Kuang C., Laskin A., Martin S. T., Ng N. L., Petaja T., Pierce J. R., Rasch P. J., Roldin P., Seinfeld J. H., Shilling J., Smith J. N., Thornton J. A., Volkamer R., Wang J., Worsnop D. R., Zaveri R. A., Zelenyuk A., Zhang Q. (2017). Recent Advances
in Understanding Secondary Organic Aerosol: Implications for Global
Climate Forcing. Rev. Geophys..

[15] Jayne J.T., Leard D. C., Zhang X., Davidovits P., Smith K. A., Kolb C. E., Worsnop D. R. (2000). Development
of an
Aerosol Mass Spectrometer for Size and Composition Analysis of Submicron
Particles Enhanced Reader. Aerosol Sci. Technol..

[16] Ng N. L., Canagaratna M. R., Jimenez J. L., Zhang Q., Ulbrich I. M., Worsnop D. R. (2011). Real-Time Methods for Estimating
Organic Component
Mass Concentrations from Aerosol Mass Spectrometer Data. Environ. Sci. Technol..

[17] Decarlo P. F., Dunlea E. J., Kimmel J. R., Aiken A. C., Sueper D., Crounse J., Wennberg P. O., Emmons L., Shinozuka Y., Clarke A., Zhou J., Tomlinson J., Collins D. R., Knapp D., Weinheimer A. J., Montzka D. D., Campos T., Jimenez J. L. (2008). Fast airborne aerosol
size and chemistry measurements above Mexico City and Central Mexico
during the MILAGRO campaign. Atmos. Chem. Phys..

[18] Harni S. D., Aurela M., Saarikoski S., Niemi J. V., Portin H., Manninen H., Leinonen V., Aalto P., Hopke P. K., Petäjä T., Rönkkö T., Timonen H. (2024). Source Apportionment
of Particle Number Size Distribution at the Street Canyon and Urban
Background Sites. Atmos. Chem. Phys..

[19] Lenschow P., Abraham H.-J., Kutzner K., Lutz M., Preub J.-D., Reichenbficher W. (2001). Some ideas
about the sources of PM10. Atmos. Environ..

[20] Sun J., Birmili W., Hermann M., Tuch T., Weinhold K., Spindler G., Schladitz A., Bastian S., Löschau G., Cyrys J., Gu J., Flentje H., Briel B., Asbach C., Kaminski H., Ries L., Sohmer R., Gerwig H., Wirtz K., Meinhardt F., Schwerin A., Bath O., Ma N., Wiedensohler A. (2019). Variability
of Black Carbon Mass Concentrations, Sub-Micrometer Particle Number
Concentrations and Size Distributions: Results of the German Ultrafine
Aerosol Network Ranging from City Street to High Alpine Locations. Atmos. Environ..

[21] Birmili, W. ; Sun, J. ; Wiedensohler, A. ; Birmili, W. ; Sun, J. ; Weinhold, K. ; Merkel, M. ; Rasch, F. ; Spindler, G. ; Wiedensohler, A. ; Bastian, S. ; Löschau, G. ; Schladitz, A. ; Quass, U. ; Kuhlbusch, T. A. J. ; Kaminski, H. ; Cyrys, J. ; Pitz, M. ; Gu, J. ; Peters, A. ; Flentje, H. ; Meinhardt, F. ; Schwerin, A. ; Bath, O. ; Ries, L. ; Gerwig, H. ; Wirtz, K. ; Weber, S. Enhanced Land Use Regression Models for Urban Fine Dust and Ultrafine Particle Concentrations View Project Radon Parallel Measurements. View Project Atmospheric Aerosol Measurements in the German Ultrafine Aerosol Network (GUAN), 2015. .

[22] Birmili W., Weinhold K., Rasch F., Sonntag A., Sun J., Merkel M., Wiedensohler A., Bastian S., Schladitz A., Löschau G., Cyrys J., Pitz M., Gu J., Kusch T., Flentje H., Quass U., Kaminski H., Kuhlbusch T. A. J., Meinhardt F., Schwerin A., Bath O., Ries L., Gerwig H., Wirtz K. (2016). Long-Term
Observations of Tropospheric Particle Number Size Distributions and
Equivalent Black Carbon Mass Concentrations in the German Ultrafine
Aerosol Network (GUAN). Earth Syst. Sci. Data.

[23] Birmili W., Wiedensohler A. (2000). New Particle
Formation in the Continental Boundary
Layer: Meteorological and Gas Phase Parameter Influence. Geophys. Res. Lett..

[24] Spindler G., Gnauk T., Grüner A., Iinuma Y., Müller K., Scheinhardt S., Herrmann H. (2012). Size-Segregated Characterization
of PM10 at the EMEP Site Melpitz (Germany) Using a Five-Stage Impactor:
A Six Year Study. J. Atmos. Chem..

[25] DeCarlo P. F., Kimmel J. R., Trimborn A., Northway M. J., Jayne J. T., Aiken A. C., Gonin M., Fuhrer K., Horvath T., Docherty K. S., Worsnop D. R., Jimenez J. L. (2006). Field-Deployable,
High-Resolution, Time-of-Flight Aerosol Mass Spectrometer. Anal. Chem..

[26] Ng N. L., Herndon S. C., Trimborn A., Canagaratna M. R., Croteau P. L., Onasch T. B., Sueper D., Worsnop D. R., Zhang Q., Sun Y. L., Jayne J. T. (2011). An Aerosol Chemical
Speciation Monitor (ACSM) for Routine Monitoring of the Composition
and Mass Concentrations of Ambient Aerosol. Aerosol Sci. Technol..

[27] Poulain L., Spindler G., Grüner A., Tuch T., Stieger B., van Pinxteren D., Petit J. E., Favez O., Herrmann H., Wiedensohler A. (2020). Multi-Year
ACSM Measurements at the Central European
Research Station Melpitz (Germany)-Part 1: Instrument Robustness,
Quality Assurance, and Impact of Upper Size Cutoff Diameter. Atmos. Meas. Tech..

[28] Atabakhsh S., Poulain L., Bigi A., Coen M. C., Pöhlker M., Herrmann H. (2025). Trends of PM1 Aerosol Chemical Composition,
Carbonaceous
Aerosol, and Source over the Last 10 Years at Melpitz (Germany). Atmos. Environ..

[29] Atabakhsh S., Poulain L., Chen G., Canonaco F., Prévôt A. S. H., Pöhlker M., Wiedensohler A., Herrmann H. (2023). A 1-Year Aerosol Chemical Speciation
Monitor (ACSM)
Source Analysis of Organic Aerosol Particle Contributions from Anthropogenic
Sources after Long-Range Transport at the TROPOS Research Station
Melpitz. Atmos. Chem. Phys..

[30] Poulain L., Spindler G., Birmili W., Plass-Dülmer C., Wiedensohler A., Herrmann H. (2011). Seasonal and Diurnal Variations of
Particulate Nitrate and Organic Matter at the IfT Research Station
Melpitz. Atmos. Chem. Phys..

[31] Spindler G., Grüner A., Müller K., Schlimper S., Herrmann H. (2013). Long-Term Size-Segregated
Particle (PM10, PM2.5, PM1)
Characterization Study at Melpitz - Influence of Air Mass Inflow,
Weather Conditions and Season. J. Atmos. Chem..

[32] Iinuma Y., Engling G., Puxbaum H., Herrmann H. (2009). A Highly Resolved Anion-Exchange
Chromatographic Method for Determination of Saccharidic Tracers for
Biomass Combustion and Primary Bio-Particles in Atmospheric Aerosol. Atmos. Environ..

[33] Canonaco F., Tobler A., Chen G., Sosedova Y., Slowik J. G., Bozzetti C., Daellenbach K. R., El Haddad I., Crippa M., Huang R. J., Furger M., Baltensperger U., Prévôt A. S. H. (2021). A New Method
for Long-Term Source
Apportionment with Time-Dependent Factor Profiles and Uncertainty
Assessment Using SoFi Pro: Application to 1 Year of Organic Aerosol
Data. Atmos. Meas. Tech..

[34] Paatero P. (1999). The Multilinear
Engine—A Table-Driven, Least Squares Program for Solving Multilinear
Problems, Including the n-Way Parallel Factor Analysis Model. J. Comput. Graph. Statist.

[35] Hammer, Ø. PAST PAleontological STatistics Reference Manual, 1999.

[36] Van
Pinxteren D., Fomba K. W., Spindler G., Müller K., Poulain L., Iinuma Y., Löschau G., Hausmann A., Herrmann H. (2016). Regional Air Quality in Leipzig,
Germany: Detailed Source Apportionment of Size-Resolved Aerosol Particles
and Comparison with the Year 2000. Faraday Discuss..

[37] Pope F. D., Gatari M., Ng’ang’a D., Poynter A., Blake R. (2018). Airborne Particulate Matter Monitoring in Kenya Using Calibrated
Low-Cost Sensors. Atmos. Chem. Phys..

[38] Petetin H., Beekmann M., Sciare J., Bressi M., Rosso A., Sanchez O., Ghersi V. (2014). A Novel Model
Evaluation Approach
Focusing on Local and Advected Contributions to Urban PM2.5 Levels
- Application to Paris, France. Geosci. Model
Dev..

[39] Gnauk T., Müller K., Brüggemann E., Birmili W., Weinhold K., Van Pinxteren D., Löschau G., Spindler G., Herrmann H. (2011). A Study to
Discriminate Local, Urban and Regional Source Contributions to the
Particulate Matter Concentrations in the City of Dresden, Germany. J. Atmos. Chem..

[40] Li G., Lei W., Bei N., Molina L. T. (2012). Contribution of
Garbage Burning to
Chloride and PM 2.5 in Mexico City. Atmos. Chem.
Phys..

[41] Huang S., Wu Z., Poulain L., Van Pinxteren M., Merkel M., Assmann D., Herrmann H., Wiedensohler A. (2018). Source Apportionment of the Organic
Aerosol over the Atlantic Ocean from 53° N to 53° S: Significant
Contributions from Marine Emissions and Long-Range Transport. Atmos. Chem. Phys..

[42] Zhang Q., Alfarra M. R., Worsnop D. R., Allan J. D., Coe H., Canagaratna M. R., Jimenez J. L. (2005). Deconvolution and
Quantification
of Hydrocarbon-like and Oxygenated Organic Aerosols Based on Aerosol
Mass Spectrometry. Environ. Sci. Technol..

[43] Wang T., Fu T., Chen K., Cheng R., Chen S., Liu J., Mei M., Li J., Xue Y. (2020). Co-Combustion Behavior of Dyeing
Sludge and Rice Husk by Using TG-MS: Thermal Conversion, Gas Evolution,
and Kinetic Analyses. Bioresour. Technol..

[44] Van Pinxteren, D. ; Mothes, F. ; Spindler, G. ; Fomba, W. ; Cuesta, A. ; Tuch, T. ; Müller, T. ; Wiedensohler, A. ; Herrmann, H. Zusatzbelastung Aus Holzheizungen; Sächsisches Landesamt für Umwelt, Landwirtschaft und Geologie, 2020.

[45] Simoneit B. R. T., Elias V. O. (2001). Detecting Organic Tracers from Biomass
Burning in the
Atmospher. Mar. Pollut. Bull..

[46] Alfarra M. R., Prevot A. S. H., Szidat S., Sandradewi J., Weimer S., Lanz V. A., Schreiber D., Mohr M., Baltensperger U. (2007). Identification of the Mass Spectral
Signature of Organic Aerosols from Wood Burning Emissions. Environ. Sci. Technol..

[47] Tobler A., Skiba A., Canonaco F., Močnik G., Rai P., Chen G., Bartyzel J., Zimnoch M., Styszko K., Nęcki J., Furger M., Różański K., Baltensperger U., Slowik J., Prévôt A. (2021). Characterization
of NR-PM_1_ and Source Apportionment of Organic Aerosol in
Krakow, Poland. Atmos. Chem. Phys..

[48] Zhang Y., Zhang X., Zhong J., Sun J., Shen X., Zhang Z., Xu W., Wang Y., Liang L., Liu Y., Hu X., He M., Pang Y., Zhao H., Ren S., Shi Z. (2022). On the Fossil
and Non-Fossil Fuel Sources of Carbonaceous
Aerosol with Radiocarbon and AMS-PMF Methods during Winter Hazy Days
in a Rural Area of North China Plain. Environ.
Res..

[49] Chen G., Sosedova Y., Canonaco F., Fröhlich R., Tobler A., Vlachou A., Daellenbach K. R., Bozzetti C., Hueglin C., Graf P., Baltensperger U., Slowik J. G., El Haddad I., Prévôt A. S. H. (2021). Time-Dependent
Source Apportionment of Submicron Organic Aerosol for a Rural Site
in an Alpine Valley Using a Rolling Positive Matrix Factorisation
(PMF) Window. Atmos. Chem. Phys..

[50] Spindler G., Brüggemann E., Gnauk T., Grüner A., Müller K., Herrmann H. (2010). A Four-Year Size-Segregated Characterization
Study of Particles PM10, PM2.5 and PM1 Depending on Air Mass Origin
at Melpitz. Atmos. Environ..

[51] Stieger B., Spindler G., Fahlbusch B., Müller K., Grüner A., Poulain L., Thöni L., Seitler E., Wallasch M., Herrmann H. (2018). Measurements of PM10
Ions and Trace Gases with the Online System MARGA at the Research
Station Melpitz in Germany – A Five-Year Study. J. Atmos. Chem..

