# Supplementary material for: Effect of Long-Range Transported Aerosol on Urban Air Quality in Eastern Germany
Source: ACS EST Air. 2025 Jul 30;2(8):1725–37. doi: 10.1021/acsestair.5c00126 (PMC12340766; doi:10.1021/acsestair.5c00126)
Supplement: Supplementary file 1 [file ea5c00126_si_001.pdf]

Effect of Long-Range Transported Aerosol on Urban Air Quality in Eastern Germany

Samira Atabakhsh[1,2], Laurent Poulain[1], Mira Pöhlker[2], Khanneh Wadinga Fomba[1], Hartmut Herrmann[1]

[1]Leibniz Institute for Tropospheric Research (TROPOS), Atmospheric Chemistry Department (ACD), Leipzig, 04318, Germany

[2]Leibniz Institute for Tropospheric Research (TROPOS), Atmospheric Microphysics Department (AMP), Leipzig, 04318, Germany

* Corresponding author: Hartmut Herrmann (herrmann@tropos.de)

1 QA/QC of instruments

To ensure the reliability and comparability of aerosol measurements across the two sites, a comprehensive set of QA/QC procedures was applied. Although a direct side-by-side intercomparison between the AMS at Melpitz and the ACSM at Eiba was not conducted during the campaign, data consistency was assessed through indirect cross-validation strategies. Both sites operated SMPS, and intercomparisons were performed within the framework of the World Calibration Centre for Aerosol Physics (WCCAP), following traceable protocols to verify the consistency of number size distributions [1].

At Melpitz, mass and volume closure assessments were performed by comparing SMPS-derived particle volume and mass concentrations, as well as off-line $PM_{2.5}$ filter measurements, with reconstructed PM_1 mass and volume from the ACSM and MAAP, applying standard density assumptions in line with [2]. At Eiba, analogous closure assessments were carried out by comparing SMPS-derived particle volume and mass, as well as filter measurements from a low-volume sampler, with AMS–MAAP-based estimates. These indirect closure evaluations, summarized in Figure S1, demonstrated strong agreement across all comparisons, supporting the completeness and internal consistency of the datasets. Together, these QA/QC procedures provide confidence in the compatibility of aerosol measurements across the rural (Melpitz) and urban (Eiba) sites, ensuring a robust foundation for the subsequent analyses presented in this study.

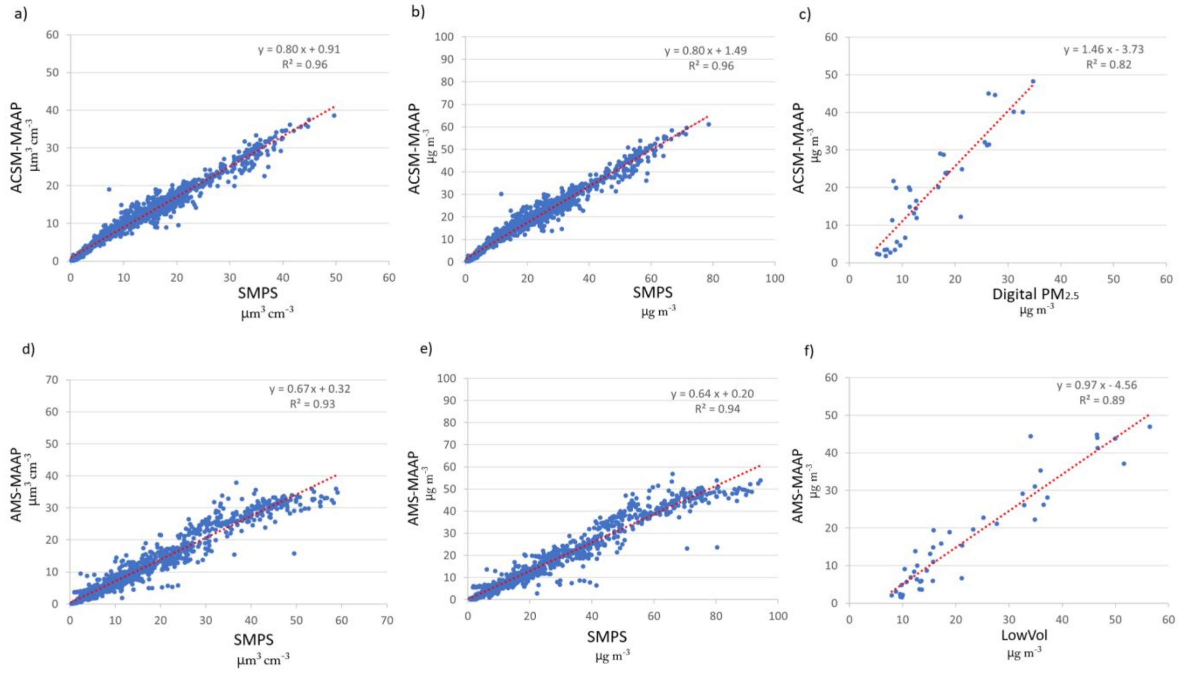

Figure S1. Comparison of ACSM–MAAP and AMS–MAAP measurements with independent particle datasets: (a–c) Melpitz station — (a) particle volume closure with SMPS-derived volume, (b) particle mass closure with SMPS-derived mass, and (c) mass closure with off-line $PM_{2.5}$ filter measurements; (d–f) Eiba station — (d) particle volume closure with SMPS-derived volume, (e) particle mass closure with SMPS-derived mass, and (f) mass closure with filter measurements from a low-volume sampler. Adapted from [2].

2 Source Apportionment of Organic Aerosol (OA) with PMF

In this study, Positive Matrix Factorization (PMF) was employed to apportion OA sources at the Eiba station during the winter 2017 campaign. The analysis followed the protocol of [3].

2.1 PMF at Eiba

2.1.1 Unconstrained PMF

As a first step, unconstrained PMF runs were conducted using factor numbers ranging from 4 to 6. Among these, the 5-factor solution provided the best compromise between residual minimization and interpretability. As shown in Figure S2, the solution yielded five distinct components. Panel (a) presents their time series over the full measurement period, while panel (b) displays the corresponding mass spectra. Based on characteristic *m/z* fragments and temporal behavior, preliminary identification of the factors suggests the presence of: Hydrocarbon-like OA (HOA) – enhanced *m/z* 41 and 57; Biomass burning OA (BBOA) – peaks at *m/z* 60 and 73; Coal combustion OA (CCOA) – notable *m/z* 115; Two oxygenated OA (OOA) factors, distinguished by their relative signal at *m/z* 43 and 44.

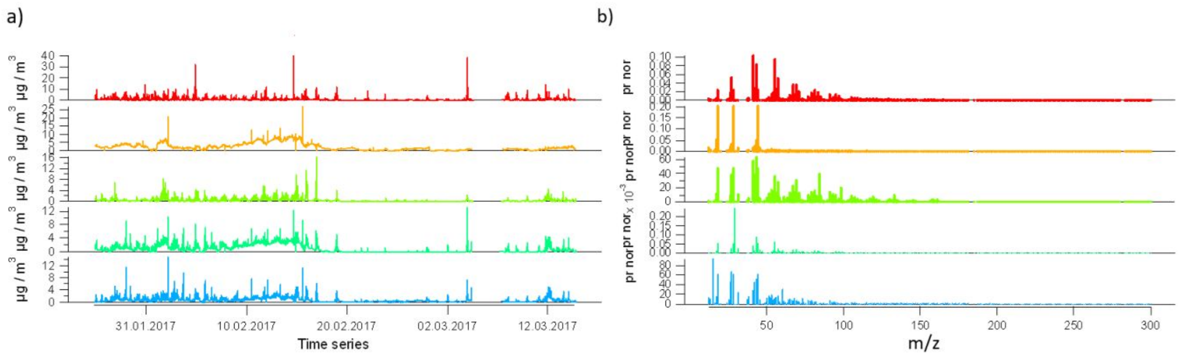

Figure S2. Five-factor unconstrained PMF solution for OA at the Eiba site. (a) Time series of the five resolved factors from the unconstrained run. (b) Corresponding unit mass resolution (UMR) spectra for each factor.

However, the profiles for primary factors were unclear, and the cooking OA (COA) factor was unresolved due to the mixing of factors. It should be mentioned that there was a tracer of coal combustion in the COA factor, as the restaurants in the area used coal for cooking. Therefore, the unconstrained solution was deemed environmentally insufficient for detailed source attribution, prompting the application of a constrained ME-2 approach, as described in Section 2.1.2.

2.1.2 Constrained rolling ME-2 Analysis

Introducing constraints based on prior knowledge is an efficient strategy for avoiding the mixing of primary factors [4]. For this reason, the ME-2 algorithm [5] enables the incorporation of time series and factor profile constraints in the form of the a-value approach. In dealing with a profile constraint, a-value specifies the variety of a factor that can deviate from the anchor profile during the PMF iteration:

$$f_{j,solution} = f_j + a \cdot f_i \quad (1)$$

To improve source resolution at Eiba, constraints were applied to three primary OA factors: HOA, BBOA, and coal combustion OA (CCOA). Constraining was done using anchor profiles which were generated from our own data during this work. We used an a-value sensitivity scan (a = 0.0–0.4 (step size: 0.1)) on HOA, BBOA and CCOA profiles to explore model flexibility. Profile-only constraints were used, and bootstrap analysis was applied to assess solution stability. The constrained model converged to a five-factor solution; HOA, BBOA, CCOA, LO-OOA, and MO-OOA.

Following the pre-test (or unconstrained) PMF, a rolling PMF with a 5-day window and a 1-day shift was applied to detect source variations. To assess statistical uncertainties, the bootstrap strategy was employed for multiple repetitions per window. This rolling PMF along with bootstrap analysis produce numerous individual PMF solutions. This necessitated the identification of environmentally reasonable solutions through user-defined criteria.

2.1.3 Source Identification Criteria

To ensure environmental relevance of each run, the constrained PMF factors were identified using the following criteria:

- Correlation of *m/z* 57 with HOA
- Correlation of levoglucosan with BBOA
- Correlation of *m/z* 115 with CCOA
- Co-variation of BBOA and CCOA with eBC(PM_1) based on R^2 values
- Classification of OOA factors using f_{44} for MO-OOA and f_{43} for LO-OOA

The final constrained profiles and time series are shown in Figure 4 and S4.

2.1.4 Unresolved OA in PMF

To evaluate the performance of the final PMF solution at the Eiba site, we assessed the difference between the measured total OA and the sum of the PMF-resolved source contributions. As illustrated in Figure S3, the sum of the resolved sources (HOA, BBOA, CCOA, LO-OOA, MO-OOA) consistently underestimates the total OA, revealing the presence of a substantial unresolved OA fraction.

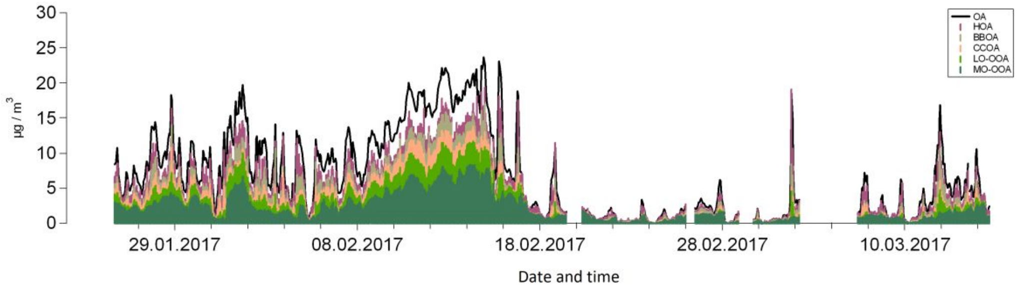

Figure S3. Time series of total organic aerosol (OA, black line) and PMF-resolved OA sources at Eiba during the winter 2017 campaign. The stacked colored areas represent five factor contributions: HOA, BBOA, CCOA, LO-OOA, and MO-OOA. The figure illustrates the temporal variability in source contributions and highlights the performance of the five-factor solution for characterizing OA composition.

On average, 23% of the measured OA mass remains unexplained by the model. This residual may arise from several factors, including limitations in the number of factors selected in the PMF solution or inherent uncertainties in the model. Despite increasing the number of factors in the PMF model for Eiba, a portion of the total OA remained unexplained, indicating that further refinements in source apportionment techniques or additional chemical information may be required to capture all contributing sources fully.

2.2 PMF at Melpitz

For the Melpitz site, PMF analysis from September 2016 to August 2017 was carried out by [6] following the standardized protocol and criteria recommended by [3]. This analysis identified five factors: three primary organic aerosols (POAs), HOA, BBOA, and CCOA, and two oxygenated OAs - LO-OOA and MO-OOA. Detailed information about processing the rolling PMF data for the one-year and ten-year datasets at Melpitz can be found in [6,7].

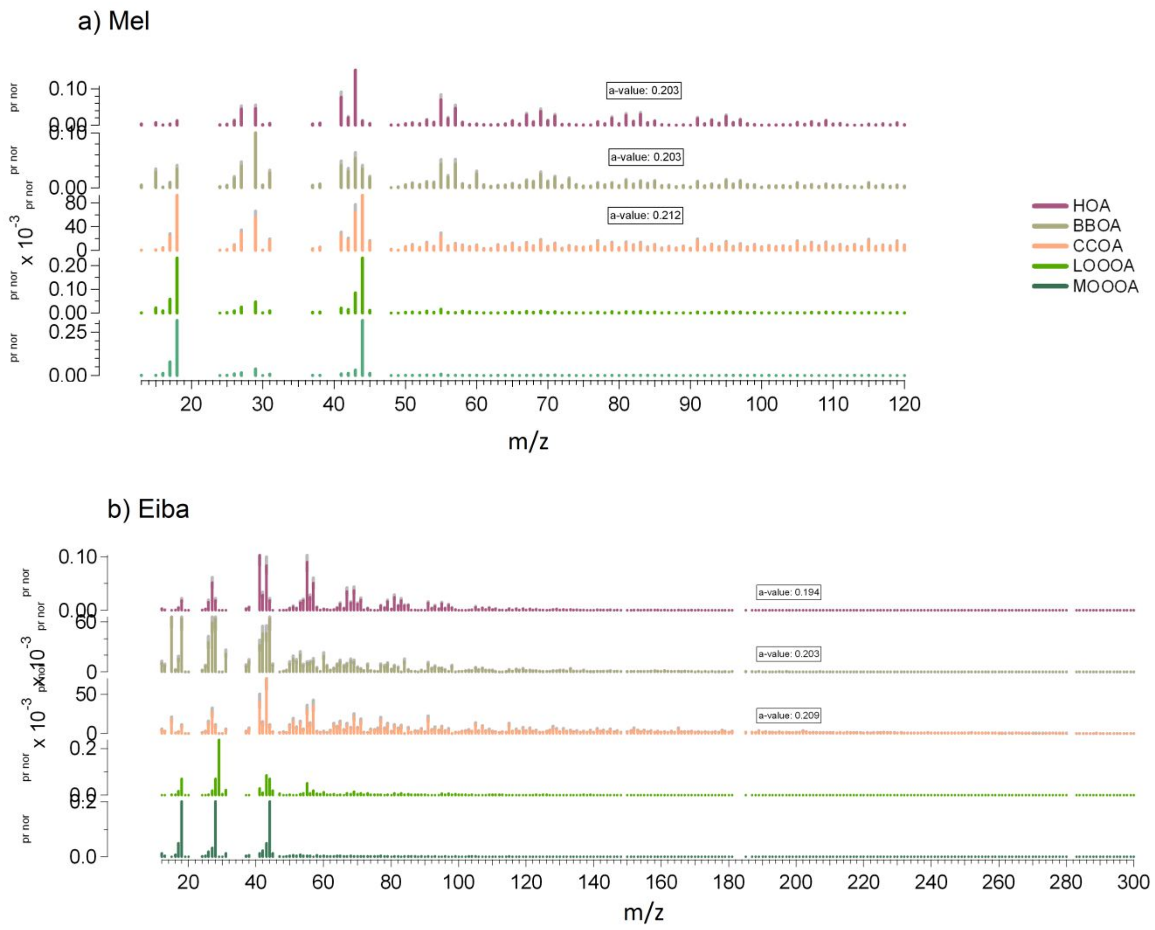

Figure S4. Mass spectral profiles of OA-PMF factors derived from ACSM and AMS measurements at (a) Melpitz and (b) Eiba. Each panel displays the unit-mass resolution spectra for the identified OA components: HOA, BBOA, CCOA, LO-OOA, and MO-OOA. The x-axis represents the mass-to-charge ratio (*m/z*) of detected ions, and the y-axis shows the relative signal intensity (normalized ion signal as a fraction of the total signal per unit *m/z*). Note that the x-axis range differs between the two panels due to instrument-specific mass detection limits: the ACSM measures up to *m/z* 120, while the AMS captures a wider range up to *m/z* 300. These average mass spectra represent the full measurement period (January–March 2017) and provide insights into the chemical composition and source characteristics of OA at both sites.

3 cross-correlation

To ensure consistency in temporal resolution between the AMS and ACSM instruments, the datasets from both locations were averaged to a uniform 1-hour timescale prior to performing the cross-correlation analysis. To maintain data integrity and ensure meaningful comparisons, only those hourly intervals with valid and overlapping measurements from both stations were included. For the Eastern-wind-period, complete data coverage was available, while 86% of the data were retained for the Western-wind-period after filtering. This harmonized approach enabled robust and reliable analysis of temporal relationships between the two sites.

Furthermore, the analysis examined time lags ranging from -20 to +20 hours to account for possible transport delays between the two sites. Positive lag values indicate that data from Eiba (urban) is shifted forward in time relative to Melpitz (regional background), suggesting that observations at Melpitz occur before those at Eiba. This pattern aligns with the predominant east-to-west transport direction. The same lag framework was used for both the East and West periods.

3.1 Impact of diurnal profile

Since cross-correlation was performed using hourly-resolved data, the influence of the diurnal cycle, linked to local emissions, temperature-driven processes, and boundary layer dynamics, could introduce noise and conceal signals related to regional or long-range transport (LRT). To reduce this meteorologically driven variability and better isolate transport-related correlations, the diurnal component was removed from the time series. The following procedure was applied:

1. The dataset was grouped by hour of the day (0–23).

2. The mean value for each hour was calculated across the entire time series.

3. These hourly means were subtracted from the corresponding hourly values in the original dataset.

The results are presented in Figures S5 and S6, where panel (a) shows the original cross-correlation and panel (b) reflects the detrended version after diurnal cycle removal.

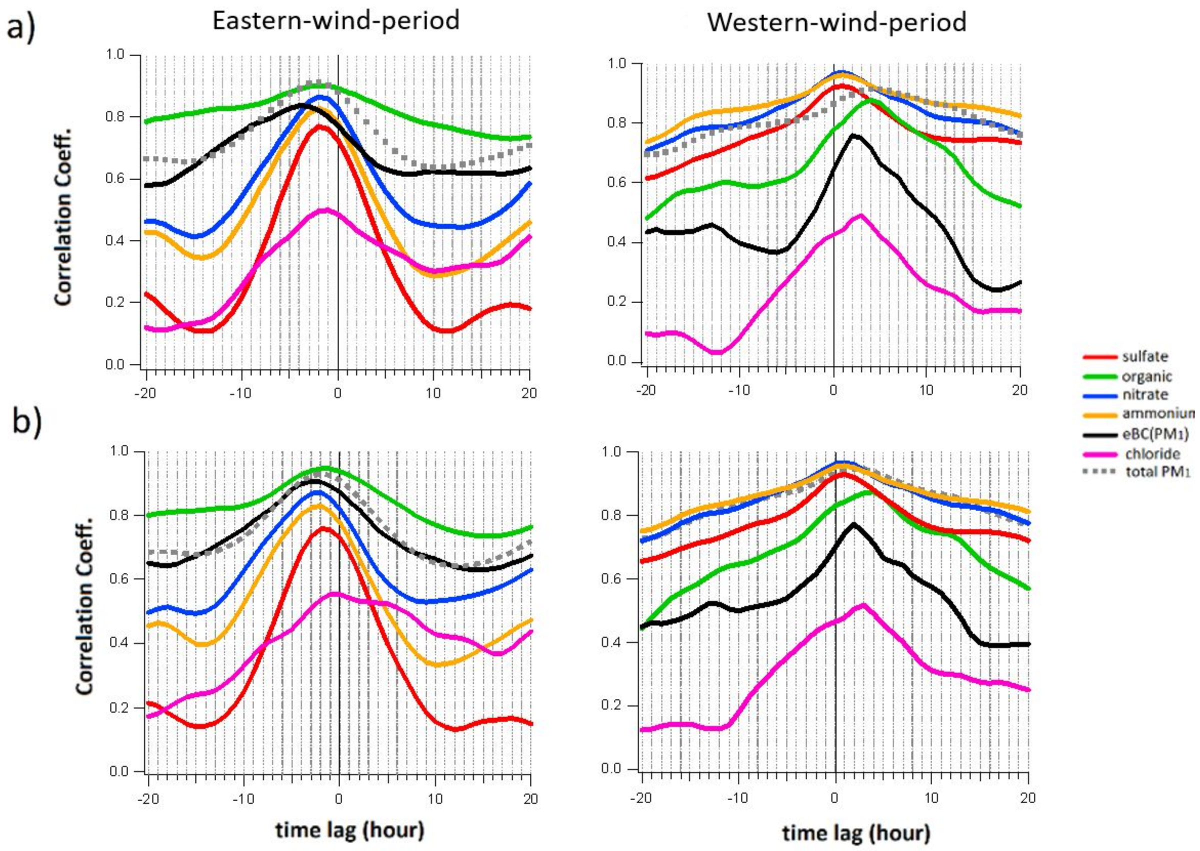

Figure S5. Cross-correlation coefficients between Melpitz and Eiba for PM_1 chemical species under Eastern (left) and Western (right) wind-periods. Panel (a) shows results using hourly-resolved data; panel (b) shows results after removing the diurnal cycle. The x-axis indicates the time lag (in hours) between the two stations.

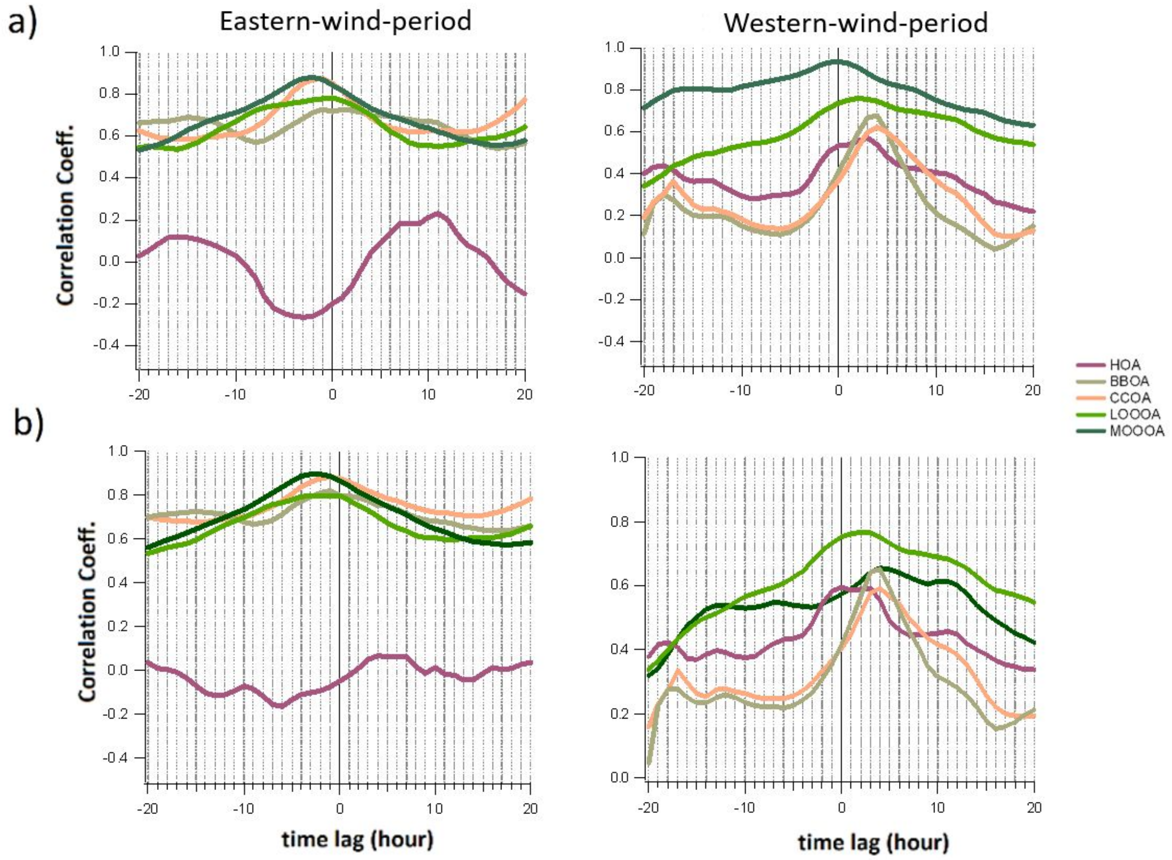

Figure S6. Cross-correlation coefficients between Melpitz and Eiba for PMF factors under Eastern (left) and Western (right) wind-periods. Panel (a) shows results using hourly-resolved data; panel (b) shows results after removing the diurnal cycle. The x-axis indicates the time lag (in hours) between the two stations.

In the Western-wind-period, removal of the diurnal cycle led to a clearer correlation for LO-OOA compared to MO-OOA, reversing the relative strength observed in the original data. This suggests that LO-OOA was more influenced by diurnal variability, with its underlying regional transport signal becoming more evident after detrending. HOA exhibited a flatter and less structured correlation profile, consistent with its attribution to local emissions governed by time-of-day activity. BBOA and CCOA showed negligible changes between the original and detrended data.

In the Eastern-wind-period, HOA displayed a reduction in short-term variability after diurnal cycle removal, indicating that high-frequency fluctuations were largely driven by local sources with strong diurnal behavior. Other PMF factors and PM_1 components maintained similar correlation patterns and time lags in both conditions.

Although moderate changes in correlation magnitude and sharpness were observed, the overall correlation structure and time shift patterns remained broadly consistent. These results confirm that the primary conclusions of the study—regarding inter-site influence and long-range transport—are robust

to the presence or removal of the diurnal cycle. This additional comparison is provided here in the Supplementary Information to ensure transparency and reproducibility of the analysis.

4 Comparison Under Similar Meteorological Conditions

To assess whether differences in pollution levels during the Eastern and Western-wind-periods were driven solely by air mass origin or also influenced by meteorological factors, we analyzed a short period from 31 January to 1 February 2017 (Case.S). This period was characterized by similar wind speed and temperature to the Western-wind-period but featured an Eastern-wind-period, in contrast to the typical westerly flow observed during clean episodes.

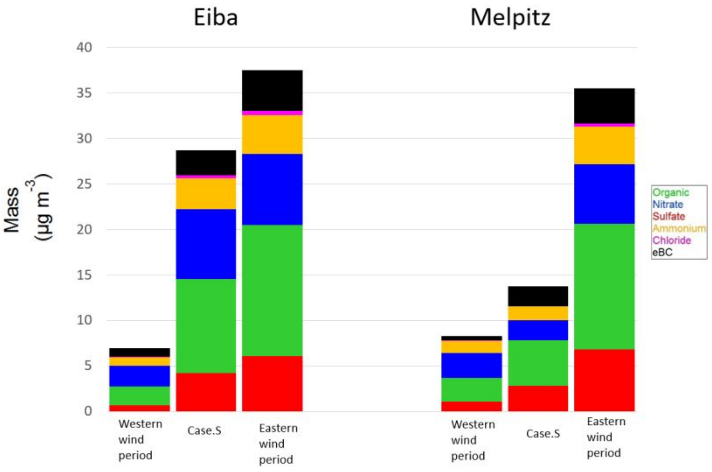

Figure S7. Average PM_1 chemical composition at Eiba and Melpitz during the Western-wind-period, Eastern-wind-period, and case study (Case.S) period. The Case.S period (30 January to 01 February 2017) was selected based on similar meteorological conditions to the Western-wind-period (e.g., temperature and wind speed) but with predominant easterly winds. Bars represent the mean mass concentrations ($\mu g\ m^{-3}$) of major PM_1 components.

As shown in Figure S7, PM_1 concentrations at both Melpitz and Eiba during Case.S were higher than during the Western-wind-period, although not as elevated as during the full Eastern-wind-period. This intermediate pattern indicates that while meteorological conditions such as temperature and wind speed influence dispersion and local accumulation, air mass direction remains a dominant factor in explaining observed concentration differences.

It is important to note that Case.S represents only two days, in contrast to the longer Eastern and Western-wind-periods used in the primary analysis. The limited duration reduces statistical representativeness and may introduce variability. Nevertheless, this case supports the robustness of our directional filtering approach and highlights the interplay between transport pathways and meteorological conditions.

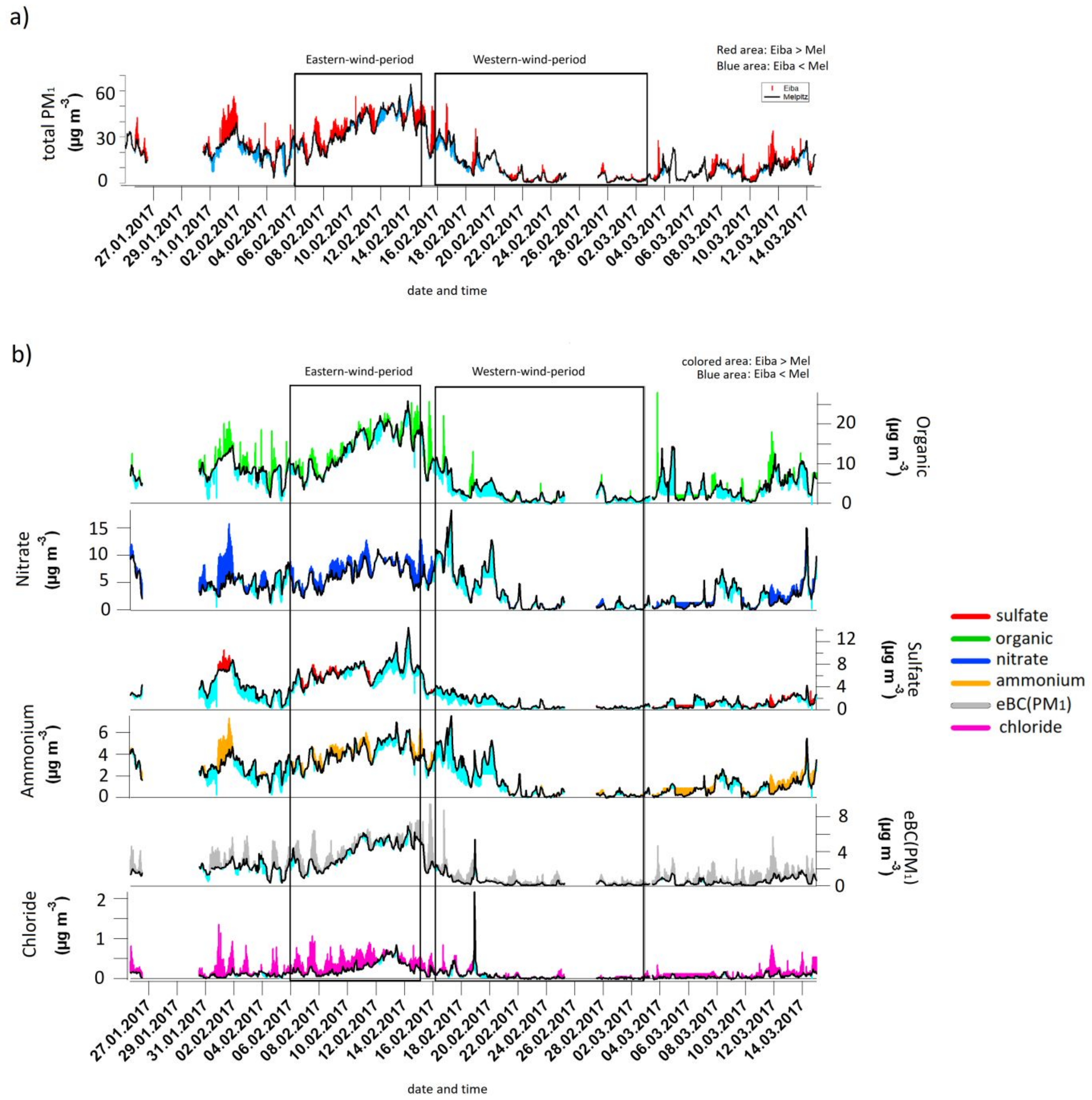

Figure S8. Time series of PM_1 mass concentrations at Melpitz and Eiba (UTC). (a) Total PM_1 concentration, with red shading indicating periods when concentrations at Eiba exceeded those at Melpitz, and blue shading indicating the opposite. (b) Mass concentrations of PM_1 species: organic aerosol, nitrate, sulfate, ammonium, equivalent black carbon (eBC(PM1)), and chloride. For each species, the black line represents Melpitz and the colored line represents Eiba. The colored line is species-colored when Eiba > Melpitz and blue when Eiba < Melpitz. Periods with persistent Eastern and Western wind-periods are marked with black boxes.

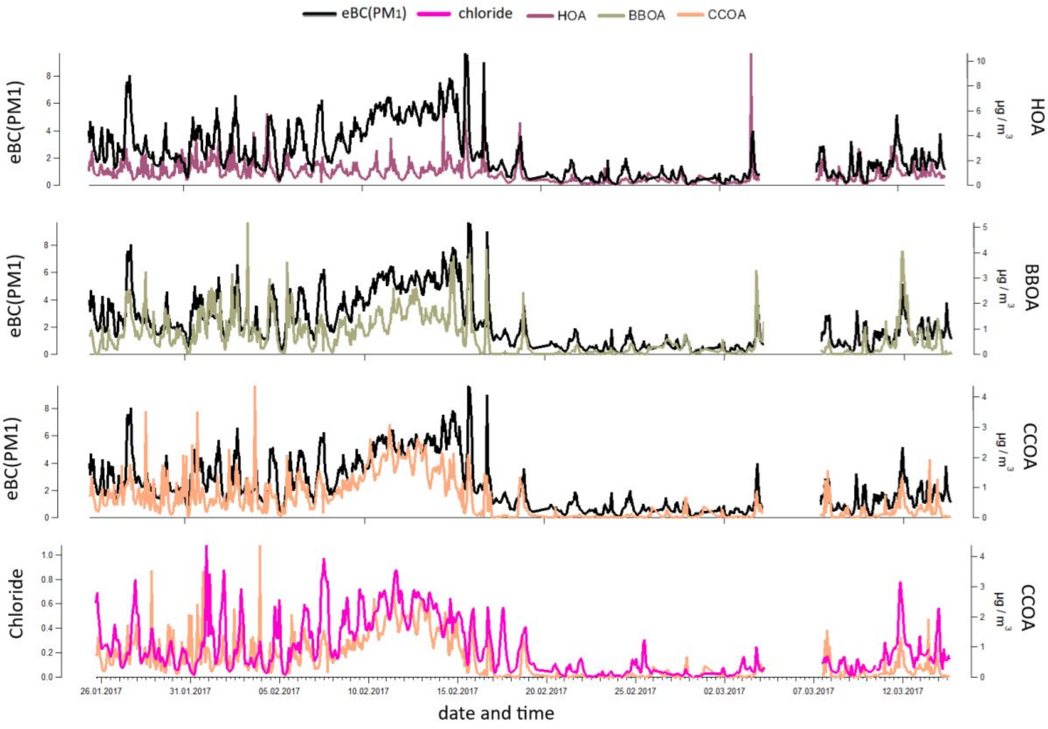

Figure S9. Time series of eBC(PM_1), chloride, and selected primary organic aerosol (OA) source factors—HOA, BBOA, and CCOA—at the Eiba site. Each panel shows eBC(PM_1) (black line) plotted alongside one or more OA factors or chloride (colored lines) over the winter 2017 measurement period.

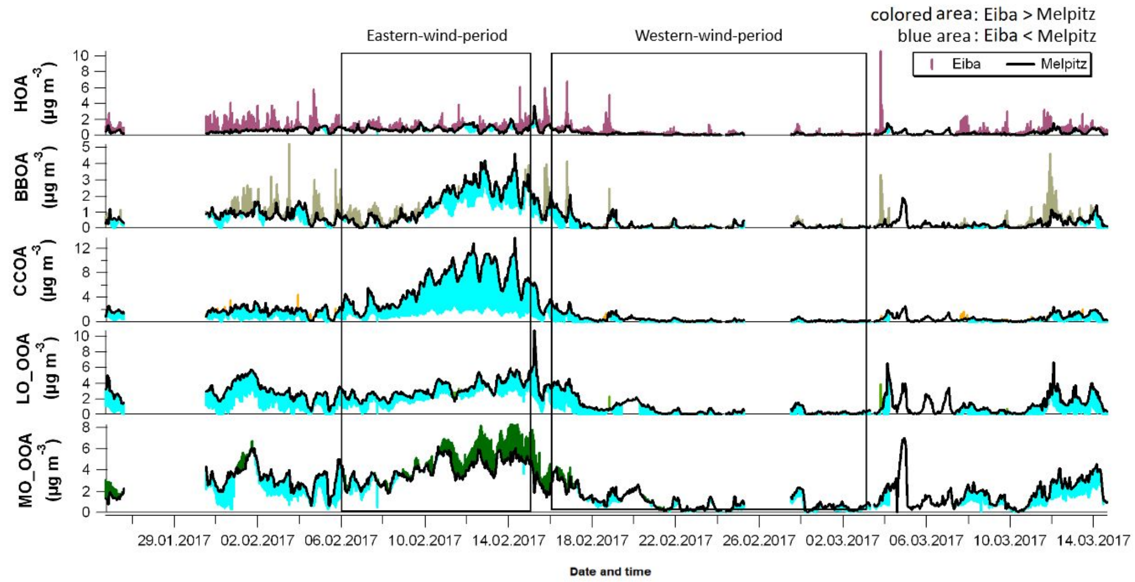

Figure S10. Comparison of PMF factor time series between the Eiba and Melpitz stations (Time is in UTC). Each panel corresponds to one OA source: HOA, BBOA, CCOA, LO-OOA, and MO-OOA. Melpitz data are shown in black, while Eiba data are overlaid in color. Blue shading indicates periods when Eiba concentrations are lower than Melpitz, whereas colored shading marks periods when Eiba concentrations exceed those at Melpitz. The "Eastern-wind-period" and "Western-wind-period" are highlighted to support the interpretation of spatial and meteorological influences on source contributions.

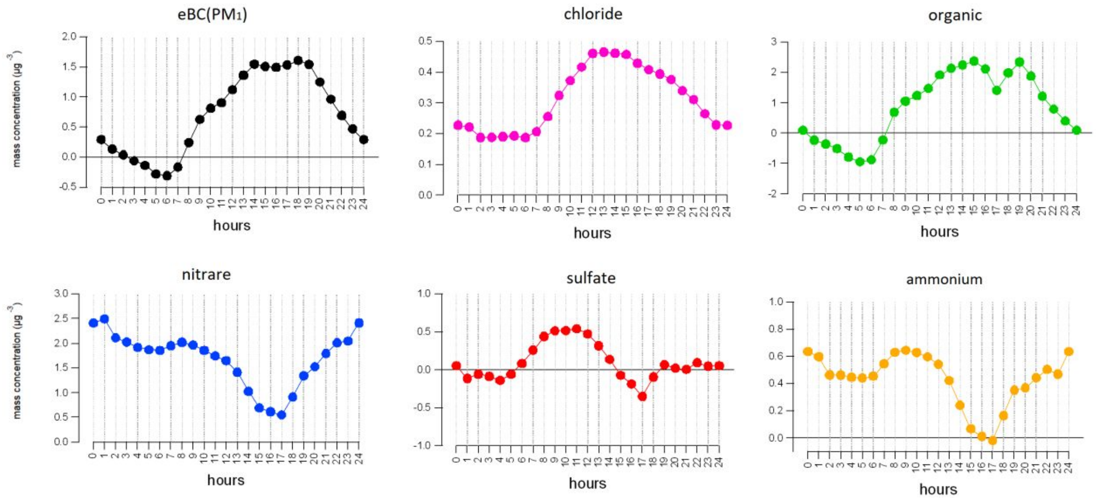

Figure S11. Diurnal profiles of the estimated urban increment (UI) for eBC(PM_1), chloride, organic, nitrate, sulfate, and ammonium at Eiba during the Eastern-wind-period under positive UI conditions.

5 Non-Parametric Wind Regression (NWR)

Non-parametric wind regression (NWR) was applied to identify the relationship between pollutant concentrations and wind direction/speed, providing insights into both local influences and transported emission sources. This method estimates the average concentration of a given species or factor for each wind vector without assuming a specific functional form, thereby capturing non-linear dependencies between concentration and wind conditions.

Following the approach described by [8], NWR was used to approximate the concentrations of PM_1 chemical species and organic aerosol (OA) sources as a function of wind direction and speed. This allowed for the visualization of dominant wind sectors associated with elevated concentrations and potential source regions. The method has previously been demonstrated as effective for identifying both local and regional contributions to observed aerosol burdens [9].

In this study, NWR plots were constructed using the full measurement period, providing a broad overview of prevailing wind-concentration relationships at both Melpitz and Eiba. It should be noted, however, that this method reflects long-term patterns and is not designed to distinguish short-term synoptic episodes or differentiate between the specific East and West transport periods investigated in the directional analyses.

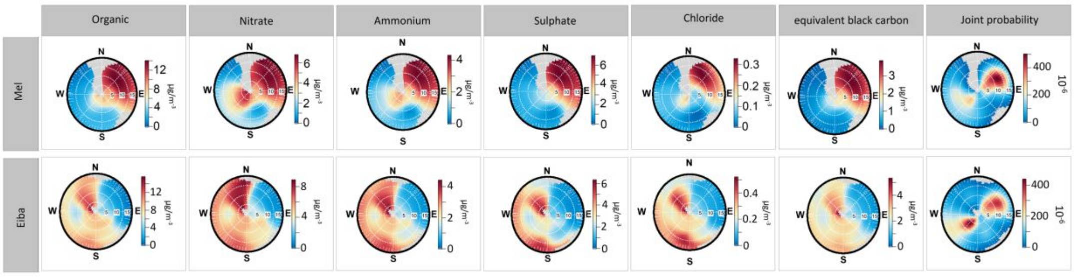

Figure S12. Non-parametric wind regression (NWR) plots for PM_1 chemical species and joint wind probability at Melpitz (top row) and Eiba (bottom row). Each polar plot shows the average concentration of a given species as a function of wind direction and wind speed (radial axis in m s^{-1}) over the full measurement period (January–March 2017). Species include organic aerosol, nitrate, ammonium, sulfate, chloride, and equivalent black carbon. The rightmost column displays joint wind probability plots, indicating the frequency distribution of wind direction and speed. These plots are based on the complete dataset and are not restricted to specific wind sectors.

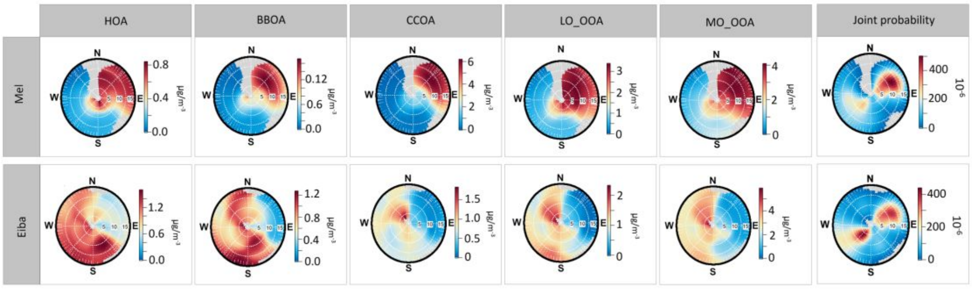

Figure S13. Non-parametric wind regression (NWR) plots PMF factors and joint wind probability at Melpitz (top row) and Eiba (bottom row). Each polar plot shows the average concentration of a given factor as a function of wind direction and wind speed (radial axis in m s^{-1}) over the full measurement period (January–March 2017). PMF factors include HOA, BBOA, CCOA, LO-OOA, and MO-OOA. The rightmost column displays joint wind probability plots, indicating the frequency distribution of wind direction and speed. These plots are based on the complete dataset and are not restricted to specific wind sectors.

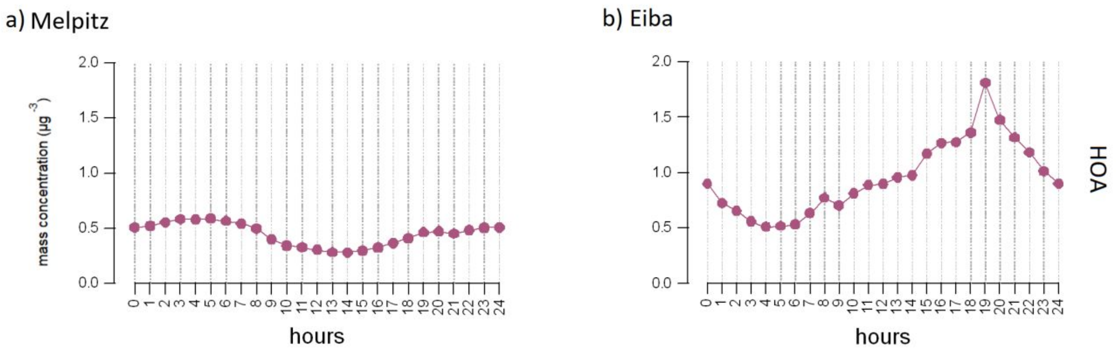

Figure S14. Diurnal profile of hydrocarbon-like organic aerosol (HOA) at (a) Melpitz and (b) Eiba over the full measurement period. The plots show hourly averaged mass concentrations (μg m^{-3}).

References

(1) Wiedensohler, A.; Birmili, W.; Nowak, A.; Sonntag, A.; Weinhold, K.; Merkel, M.; Wehner, B.; Tuch, T.; Pfeifer, S.; Fiebig, M.; Fjäraa, A. M.; Asmi, E.; Sellegri, K.; Depuy, R.; Venzac, H.; Villani, P.; Laj, P.; Aalto, P.; Ogren, J. A.; Swietlicki, E.; Williams, P.; Roldin, P.; Quincey, P.; Hüglin, C.; Fierz-Schmidhauser, R.; Gysel, M.; Weingartner, E.; Riccobono, F.; Santos, S.; Grüning, C.; Faloon, K.; Beddows, D.; Harrison, R.; Monahan, C.; Jennings, S. G.; O'Dowd, C. D.; Marinoni, A.; Horn, H. G.; Keck, L.; Jiang, J.; Scheckman, J.; McMurry, P. H.; Deng, Z.; Zhao, C. S.; Moerman, M.; Henzing, B.; De Leeuw, G.; Löschau, G.; Bastian, S. Mobility Particle Size Spectrometers: Harmonization of Technical Standards and Data Structure to Facilitate High Quality Long-Term Observations of Atmospheric Particle Number Size Distributions. *Atmos Meas Tech* **2012**, *5* (3), 657–685. https://doi.org/10.5194/amt-5-657-2012.

(2) Poulain, L.; Spindler, G.; Grüner, A.; Tuch, T.; Stieger, B.; Pinxteren, D. Van; Petit, J. E.; Favez, O.; Herrmann, H.; Wiedensohler, A. Multi-Year ACSM Measurements at the Central European Research Station Melpitz (Germany)-Part 1: Instrument Robustness, Quality Assurance, and Impact of Upper Size Cutoff Diameter. *Atmos Meas Tech* **2020**, *13* (9), 4973–4994. https://doi.org/10.5194/amt-13-4973-2020.

(3) Chen, G.; Canonaco, F.; Tobler, A.; Aas, W.; Alastuey, A.; Allan, J.; Atabakhsh, S.; Aurela, M.; Baltensperger, U.; Bougiatioti, A.; De Brito, J. F.; Ceburnis, D.; Chazeau, B.; Chebaicheb, H.; Daellenbach, K. R.; Ehn, M.; El Haddad, I.; Eleftheriadis, K.; Favez, O.; Flentje, H.; Font, A.; Fossum, K.; Freney, E.; Gini, M.; Green, D. C.; Heikkinen, L.; Herrmann, H.; Kalogridis, A. C.; Keernik, H.; Lhotka, R.; Lin, C.; Lunder, C.; Maasikmets, M.; Manousakas, M. I.; Marchand, N.; Marin, C.; Marmureanu, L.; Mihalopoulos, N.; Močnik, G.; Nęcki, J.; O'Dowd, C.; Ovadnevaite, J.; Peter, T.; Petit, J. E.; Pikridas, M.; Matthew Platt, S.; Pokorná, P.; Poulain, L.; Priestman, M.; Riffault, V.; Rinaldi, M.; Różański, K.; Schwarz, J.; Sciare, J.; Simon, L.; Skiba, A.; Slowik, J. G.; Sosedova, Y.; Stavroulas, I.; Styszko, K.; Teinemaa, E.; Timonen, H.; Tremper, A.; Vasilescu, J.; Via, M.; Vodička, P.; Wiedensohler, A.; Zografou, O.; Cruz Minguillón, M.; Prévôt, A. S. H. European Aerosol Phenomenology − 8: Harmonised Source Apportionment of Organic Aerosol Using 22 Year-Long ACSM/AMS Datasets. *Environ Int* **2022**, *166*. https://doi.org/10.1016/j.envint.2022.107325.

(4) Crippa, M.; Canonaco, F.; Lanz, V. A.; Äijälä, M.; Allan, J. D.; Carbone, S.; Capes, G.; Ceburnis, D.; Dall'Osto, M.; Day, D. A.; DeCarlo, P. F.; Ehn, M.; Eriksson, A.; Freney, E.; Ruiz, L. H.; Hillamo, R.; Jimenez, J. L.; Junninen, H.; Kiendler-Scharr, A.; Kortelainen, A. M.; Kulmala, M.; Laaksonen, A.; Mensah, A. A.; Mohr, C.; Nemitz, E.; O'Dowd, C.; Ovadnevaite, J.; Pandis, S. N.; Petäjä, T.; Poulain, L.; Saarikoski, S.; Sellegri, K.; Swietlicki, E.; Tiitta, P.; Worsnop, D. R.; Baltensperger, U.; Prévôt, A. S. H. Organic Aerosol Components Derived from 25 AMS Data Sets across Europe Using a Consistent ME-2 Based Source Apportionment Approach. *Atmos Chem Phys* **2014**, *14* (12), 6159–6176. https://doi.org/10.5194/acp-14-6159-2014.

(5) Paatero, P. The Multilinear Engine—A Table-Driven, Least Squares Program for Solving Multilinear Problems, Including the n-Way Parallel Factor Analysis Model. *Journal of Computational and Graphical Statistics* **1999**, *8* (4), 854–888. https://doi.org/10.1080/10618600.1999.10474853.

(6) Atabakhsh, S.; Poulain, L.; Chen, G.; Canonaco, F.; Prévôt, A. S. H.; Pöhlker, M.; Wiedensohler, A.; Herrmann, H. A 1-Year Aerosol Chemical Speciation Monitor (ACSM) Source Analysis of Organic Aerosol Particle Contributions from Anthropogenic Sources after Long-Range Transport at the TROPOS Research Station Melpitz. *Atmos Chem Phys* **2023**, *23* (12), 6963–6988. https://doi.org/10.5194/acp-23-6963-2023.

(7) Atabakhsh, S.; Poulain, L.; Bigi, A.; Coen, M. C.; Pöhlker, M.; Herrmann, H. Trends of PM1 Aerosol Chemical Composition, Carbonaceous Aerosol, and Source over the Last 10 Years at Melpitz (Germany). *Atmos Environ* **2025**, *346*. https://doi.org/10.1016/j.atmosenv.2025.121075.

(8) Henry, R.; Norris, G. A.; Vedantham, R.; Turner, J. R. Source Region Identification Using Kernel Smoothing. *Environ Sci Technol* **2009**, *43* (11), 4090–4097. https://doi.org/10.1021/es8011723.

(9) Marin, C. A.; Mărmureanu, L.; Radu, C.; Dandocsi, A.; Stan, C.; Ţoancă, F.; Preda, L.; Antonescu, B. Wintertime Variations of Gaseous Atmospheric Constituents in Bucharest Peri-Urban Area. *Atmosphere (Basel)* **2019**, *10* (8). https://doi.org/10.3390/atmos10080478.
